# Neuronal and nonneuronal TRPA1 mediate distinct pain perception mechanisms in Familial Episodic Pain Syndrome models

**DOI:** 10.1172/JCI206667

**Published:** 2026-10-01

**Authors:** Matilde Marini, Martina Chieca, Elisabetta Coppi, Marco Montini, Lorenzo Bonacchi, Lorenzo Landini, Irene Scuffi, Kelvin Y. Kwan, Alice Papini, Gaetano De Siena, Elisa Bellantoni, Lucrezia Timotei, Elena Colzi, Valentina Albanese, Gabriele Ferroni, Evelynn D.N. Melo, Marie-Christine Birling, Romain Lorentz, Romina Nassini, Francesco De Logu

**Affiliations:** 1 Department of Health Sciences, Clinical Pharmacology and Oncology Section,; 2 Department of Neuroscience, Psychology, Drug Research and Child Health (NEUROFARBA), Section of Pharmacology and Toxicology, and; 3 Department of Experimental and Clinical Biomedical Sciences, University of Florence, Florence, Italy.; 4 Department of Cell Biology & Neuroscience, Keck Center for Collaborative Neuroscience, Stem Cell Research Center, Rutgers University, Piscataway, New Jersey, USA.; 5 Department of Chemical, Pharmaceutical and Agricultural Sciences, University of Ferrara, Ferrara, Italy.; 6 Laboratory of Bioactive Products, Federal University of Rio de Janeiro, Multidisciplinary Centre UFRJ-Macaé, Macaé, Brazil.; 7 CNRS, INSERM, CELPHEDIA, PHENOMIN, Institut Clinique de la Souris, UAR2062 – PHEN-ICS, France.

**Keywords:** Genetics, Inflammation, Neuroscience, Genetic diseases, Ion channels, Mouse models

## Abstract

Familial Episodic Pain Syndrome (FEPS) is a rare inherited disorder characterized by recurrent episodes of severe upper-body pain triggered by stimuli such as cold, stress, or fasting. A gain-of-function mutation (N855S) in the Transient Receptor Potential Ankyrin 1 (TRPA1) ion channel has been identified in affected individuals, yet the cellular mechanisms driving FEPS pain remain poorly defined. Although TRPA1 has been primarily studied in sensory neurons, emerging evidence suggests that nonneuronal cells may contribute to pain signaling. To define the cell-specific role of mutant TRPA1 (TRPA1^mut^), we generated mouse models selectively expressing TRPA1^mut^ in Schwann cells or sensory neurons, using CRISPR-based and Cre-loxP strategies. Patch-clamp recordings confirmed enhanced agonist-evoked currents in TRPA1^mut^ compared with WT channels. Behavioral analyses revealed that neuronal TRPA1^mut^ mediates acute nociceptive responses, whereas Schwann cell TRPA1^mut^ drives mechanical allodynia induced by subthreshold TRPA1 agonists and physiological triggers relevant to FEPS, including fasting, cold exposure, and restraint stress. These responses were associated with increased reactive oxygen species and accumulation of 4-hydroxynonenal in sciatic nerves and were attenuated by antioxidant treatment. These findings support a Schwann cell–dependent mechanism of TRPA1-driven pain and identify nonneuronal TRPA1 signaling as a potential therapeutic target in FEPS and related pain channelopathies.

## Abstract 2

A rare genetic mutation causes pain through two different cell types, revealing an unexpected role for TRPA1 Schwann cells and pointing to more precise treatments.

## Introduction

Inherited mutations in ion channels can disrupt normal ion flux across the plasma membrane, leading to a range of conditions, including pathological pain (1–3). Nonfunctional mutations in voltage-gated sodium channels (Nav) have been linked to a lack of pain sensation in congenital indifference to pain syndrome (4–9). Conversely, missense mutations in Nav channels have been associated with severe pain episodes, such as those observed in primary erythromelalgia syndrome (10). Similarly, mutations in voltage-gated calcium channels (Cav) have been linked to conditions such as familial hemiplegic migraine (11–13) and trigeminal neuralgia (14) and to susceptibility to malignant hyperthermia (15).

The Transient Receptor Potential (TRP) family of channels includes multimodal ion channels responding to a wide range of physical and chemical stimuli, including changes in temperature, mechanical pressure, and various chemical irritants (16–20). Mutations in several TRP channels have been correlated with distinct channelopathies, including TRPC6 with focal segmental glomerulosclerosis (21); TRPV4 with autosomal dominant metatropic dysplasia and Charcot-Marie-Tooth disease type 2C (22, 23); TRPM1 with autosomal recessive congenital stationary night blindness (24); TRPML1 with mucolipidosis type IV(25); and TRPP2 with autosomal dominant polycystic kidney disease (26). However, the only known inherited human pain–associated TRP channelopathy involves a mutation in the TRPA1 subtype and is called Familial Episodic Pain Syndrome (FEPS) (2). A gain-of-function point mutation (N855S) in the S4 domain of TRPA1 was first identified in a South American family. The altered biophysical properties caused by TRPA1 channel mutation resulted in episodes of severe pain in the upper body triggered by conditions of food restriction, cold exposure, and stress. Moreover, it enhances the sensitization of the nociceptive system, as demonstrated by exaggerated and prolonged hyperalgesia and allodynia following application of the TRPA1-selective agonist mustard oil (27).

Conventionally, pain has been attributed to the activation/sensitization of sensory neurons, which detect noxious stimuli and transmit a pain signal to the central nervous system. However, recent discoveries highlight the complex interaction between neuronal and nonneuronal cells in pain development and maintenance (28–34). Schwann cells, known for their role in protecting and supporting peripheral nerves, have recently been recognized for their role in mediating mechanical and pressure sensation in the skin (35–37) and in development and maintenance of mechanical allodynia in different mouse models of pain (38–46). Here, by comparing the WT (TRPA1) and mutated (TRPA1^mut^) mouse ion channels, we confirmed that the FEPS gain-of-function point mutation altered the channel biophysical properties, with the TRPA1^mut^ channel exhibiting larger current than the outward-rectifying WT channel. In addition, using 3 distinct genetic approaches, we developed a robust FEPS mouse model with cell-selective expression of TRPA1^mut^ in sensory neurons (*Adv*-TRPA1^mut^ mice) or Schwann cells (*Plp*-TRPA1^mut^ mice), in which we identified distinct roles for TRPA1 in mediating acute nociception and mechanical allodynia, respectively. To dissect the role of neuronal and nonneuronal cells in FEPS, we also used 3 behavioral procedures in mice (food deprivation, stress induction, and cold temperature exposure) that reflect the primary triggers of body pain in patients with FEPS. We reported that *Plp*-TRPA1^mut^ but not *Adv-*TRPA1^mut^ mice were sensitive to the development of mechanical allodynia following fasting, stress, and cold-temperature exposure. The feed-forward mechanism activated by TRPA1^mut^ in Schwann cells by a subthreshold dose of reactive oxygen species (ROS) facilitated and sustained mechanical allodynia in *Plp-*TRPA1^mut^.

Our study provides evidence that neuronal and nonneuronal TRPA1 mediates distinct mechanisms of pain perception in mouse models of FEPS, with neuronal TRPA1 primarily mediating acute nociception. At the same time, Schwann cell TRPA1 is essential for sustaining mechanical allodynia. Overall, the presented FEPS mouse model provides a valuable tool for investigating the distinct roles of neuronal and Schwann cells in pain pathways, supporting the development of targeted therapeutic strategies for chronic pain.

## Results

### Conservation of the N855S mutation and biophysical characterization of mouse TRPA1^mut^.

FEPS is caused by an A-to-G transition in exon 22 of the *TRPA1* cDNA (c.A2564G), which results in the substitution of an asparagine by a serine (N855S) in the transmembrane segment S4 (27). To establish a relevant mouse model for in vivo functional studies, we first confirmed that the human mutation site region was conserved in the mouse *Trpa1* (Figure 1A). We next characterized the biophysical properties of the WT (TRPA1) and mutated mouse TRPA1 (TRPA1^mut^) channels. Plasmids encoding either TRPA1 or TRPA1^mut^ were expressed in HEK293T cells and analyzed using whole-cell patch-clamp recordings. Consistent with findings in human TRPA1(3), the overall voltage-dependent currents activated by a depolarizing voltage ramp protocol (–100 to +100 mV) in TRPA1 and TRPA1^mut^ cells were identical in absence of TRPA1 agonists (Figure 1B). However, upon stimulation with selective exogenous or endogenous TRPA1 agonists, allyl isothiocyanate (AITC) or 4-hydroxynonenal (4-HNE), respectively (both 100 μM; 2 min application), TRPA1^mut^ channel mediated markedly larger currents compared with the TRPA1 WT counterpart (Figure 1, C–J). Indeed, averaged TRPA1^mut^-activated currents, obtained by subtraction of the control ramp from that recorded in the presence of AITC (Figure 1E) or 4-HNE (Figure 1I), were at least doubled in the outward range up to +100 mV, whereas the inward component, negligible in TRPA1-transfected cells upon agonist exposure (Figure 1, E and I), was prominent. Both channels were inhibited by the selective TRPA1 antagonist A967079 (Figure 1, C, D, G, and H). These results indicate that the conserved N858S mutation in mouse TRPA1 reproduces the gain-of-function effect described in human TRPA1(27), consistent with FEPS pathogenesis and provide a basis for generating mouse models of the disease.

### Generation of mouse models carrying the TRPA1^mut^ mutation.

We recently observed that the Schwann cell (SC) and neuronal TRPA1 channel play a critical and distinct role in several mouse model of pain, including neuropathic and inflammatory pain and cancer and migraine pain (39, 40, 42, 44, 45, 47, 48). Here, we used the gain-of-function TRPA1 channel with altered biophysical properties to examine the roles of SC- and neuronal-mediated TRPA1 in a mouse model of FEPS. To investigate the physio/pathological role of the TRPA1^mut^ channel in pain, we used an adeno-associated viral (AAV) vector–based strategy to supplement functionally mutated copies of the WT *Trpa1* gene in mice. We developed 2 complementary approaches, both based on a double AAV platform that combines CRISPR-Cas9 genome editing with microhomology-mediated end-joining (MMEJ) (49) to achieve targeted expression of *Trpa1^mut^* in vivo in mice. Both platforms involve AAV-mediated, Cre-dependent expression of a loxP-flanked *Staphylococcus aureus* Cas9 (SaCas9) and eGFP (Figure 2, A and B). In the first strategy, termed genome-editing approach (GEdit), SaCas9 and 2 gRNAs excised the endogenous *Trpa1* sequence from the mouse genome of *Cre*-expressing mice and the exogenous *Trpa1^mut^* sequence from the AAV vector (*Plp*-*Cre*-GEdit mice for Schwann cells and *Adv*-Cre-GEdit mice for sensory neurons) (Figure 2A). AAVrh10 (intravenous administration) or AAV2/9n (intrathecal administration) were used in *Plp*-Cre or *Adv*-Cre mice, respectively (44, 50). The *Trpa1^mut^* sequence was then integrated in the genome using microhomology arms (MHA) with single-base-pair substitutions introduced at the gRNA recognition sites to prevent repeated cleavage (Figure 2A). The second strategy, termed minigene approach (MiniG), was developed as a complementary method. Here, we used Cre-*Trpa1^fl/fl^* mice generated by crossing cell type–specific Cre-driver lines with *Trpa1*-*fl* mice (*Trpa1^fl/fl^*, exons 22–24 flanked by *loxP* sites) (Figure 2B). Following Cre-mediated excision of the WT Trpa1 exon 22–24, an AAV vector delivers the gRNAs along with a mutated *Trpa1^mut^* minigene (exons 22–24), allowing targeted reexpression of the modified sequence (*Plp*-Cre-MiniG and *Adv*-Cre-MiniG mice). The tissue-specific effects of TRPA1^mut^ were investigated in SCs and primary sensory neurons derived from *Plp-Cre-GEdit, Adv-Cre-GEdit, Plp-Cre-MiniG,* and *Adv-Cre-MiniG*. Altered TRPA1 currents were observed in SCs from *Plp-Cre-GEdit* and *Plp-Cre-MiniG* mice, but not in SCs from *Adv-Cre-GEdit* and *Adv-Cre-MiniG* mice. Conversely, primary sensory neurons exhibited altered TRPA1 currents only in *Adv-Cre-GEdit* and *Adv-Cre-MiniG* mice (Supplemental Figure 1, A and B). A semiquantitative delivery efficiency of SaCas9 in GEdit and MiniG mice was confirmed by IHC, detecting GFP fluorescence (Supplemental Figure 2, A and B). Given that the AAVrh10 serotype has been reported to cross the blood brain barrier (BBB) (51), and considering that *Plp-Cre* may potentially drive TRPA1 editing also in oligodendrocytes (52), we next assessed whether the fluorescent reporter gene (GFP) was detectable within the central nervous system (CNS). IHC analysis of the spinal cord showed no detectable GFP fluorescence and no colocalization with the oligodendrocyte marker Olig2 (Supplemental Figure 2C). The presence of the single point mutation was confirmed by RNA long-reads sequencing in sciatic nerves tissue homogenates, (Supplemental Figure 2, D and E). Notably, despite the relatively low fraction of mutant reads (5.8% and 6.3% in *Plp-Cre-GEdit* and *Plp-Cre-MiniG* mice, respectively) detected in bulk sciatic nerve samples, this value likely underestimates the effective editing/replacement efficiency within the relevant target cell populations. In GEdit and MiniG models, editing is restricted to cells that both express Cre and are transduced by AAV, inherently limiting the detectable abundance of the mutant allele in whole-tissue analyses.

### Schwann cell and neuronal-TRPA1^mut^ differentially mediate mechanical allodynia and acute nociception.

We previously reported that the intraplantar (i.pl.) administration of AITC, the selective TRPA1 agonist, elicited both acute nociception and paw mechanical allodynia, which TRPA1 differentially mediated and expressed in neurons and SCs, respectively (53, 54). To further dissect the contribution of SC and neuronal TRPA1^mut^ in the mouse models of FEPS, we employed the i.pl. injection of the 2 selective TRPA1 agonists, AITC and 4-HNE.

Since patients with FEPS exhibit enhanced sensitization of the nociceptive system upon stimulation (27), we first identified subthreshold doses of both agonists, which elicited acute nociception and paw mechanical allodynia in FEPS mice but not in control mice. To this end, control mice were used to establish a dose-response curve for both AITC and 4-HNE (Figure 3, A and B). We found that administering a subthreshold dose of AITC (0.1 nmol) and 4-HNE (0.5 nmol) induced mechanical allodynia in *Plp*-Cre-GEdit and *Plp-*Cre-MiniG mice, but not in *Adv*-Cre-GEdit, *Adv*-Cre-MiniG, or control mice (Figure 3, C and D, and Supplemental Figure 3, A and B). This allodynia was abolished by systemic administration of the TRPA1 antagonist A967079 (Figure 3E and Supplemental Figure 3C). Conversely, the same subthreshold dose of AITC and 4-HNE evoked an acute nociceptive response in *Adv*-Cre-GEdit and *Adv*-Cre-MiniG mice, but not in *Plp*-Cre-GEdit, *Plp*-Cre-MiniG, or control mice (Figure 3, F and G, and Supplemental Figure 3, D and E). The nociceptive response observed in *Adv*-Cre-GEdit and *Adv*-Cre-MiniG mice was blocked by A967079 (Figure 3H and Supplemental Figure 3F). Collectively, these data reveal that the FEPS-associated TRPA1 mutation in SCs predominantly drives mechanical allodynia. In contrast, the mutation in sensory neurons underlies enhanced acute nociceptive responses.

### Conditional knock-in model confirms dichotomous roles of SC and neuronal TRPA1^mut^.

To further validate the distinct roles of SCs and sensory neurons in FEPS, we designed a conditional knock-in (cKI) mouse model expressing the heterozygous FEPS mutation. The cKI-TRPA1 mouse model was created using an advanced Cre-dependent genetic switch strategy (55). This Cre-*loxP* technology-based method enables a shift in genetic expression from the WT mouse TRPA1 to the mutated TRPA1^mut^. To generate the cKI-TRPA1^mut^ mouse, we constructed a plasmid to electroporate C57BL/6N embryonic stem (ES) cells, containing *loxP*-flanked DNA sequence from mouse exon 22 in the sense orientation and the mutated and degenerated exon 22 in the antisense orientation (Figure 4A and Supplemental Figure 4, A–H). Following selective Cre recombination, the mouse WT exon was excised, and the mutated exon was inverted to produce *Plp*-cKI-*Trpa1^mut^* and *Adv*-cKI-*Trpa1^mut^* mice that harbor mutant *Trpa1^mut^* exon specifically in SCs and sensory neurons, respectively (Figure 4A).

Subthreshold dose (i.pl.) of AITC and 4-HNE triggered mechanical allodynia in *Plp*-cKI-*Trpa1^mut^* mice but not in *Adv*-cKI- *Trpa1^mut^* or control mice (Figure 4, B and C). Conversely, the same agonists induced nociceptive behavior in *Adv*-cKI *Trpa1^mut^* mice but not in *Plp*-cKI- *Trpa1^mut^* or control mice (Figure 4, D and E). The point mutation harbored by Plp-cKI- *Trpa1^mut^* and *Adv*-cKI- *Trpa1^mut^* mice was confirmed by next-generation sequencing (Supplemental Figure 4I). The tissue-specific effects of TRPA1^mut^ were investigated in primary sensory neurons and SCs derived from *Plp*-cKI- *Trpa1^mut^, Adv*-cKI- *Trpa1^mut^,* and control mice. Altered TRPA1 currents were observed in SCs from *Plp*-cKI- *Trpa1^mut^* but not in *Adv*-cKI- *Trpa1^mut^* or control mice. Conversely, primary sensory neurons exhibited altered TRPA1 currents only in *Adv*-cKI- *Trpa1^mut^* mice (Supplemental Figure 4, J and K).

These findings confirm the dichotomous roles of SCs and neuronal TRPA1 in mediating mechanical allodynia and nociception, respectively. Importantly, the study established transgenic mouse models to investigate FEPS and TRPA1-related pain syndromes.

### Fasting, cold, and stress exposure trigger Schwann cell TRPA1-dependent allodynia in FEPS mice.

Individuals with FEPS experience recurrent episodes of severe upper body pain that begin in infancy and persist throughout life. These painful crises are notably precipitated by physiological or environmental stressors such as prolonged food restriction, emotional or physical stress, or exposure to low temperatures (27). To investigate the mechanisms and specific cells involved in pain episodes in FEPS, we tested the mechanical allodynia in transgenic mice under physiological and environmental stressors, including fasting, cold exposure, and restraint stress (56–59).

Firstly, we found that neither 24 nor 48 hours of food deprivation induced mechanical allodynia in control mice (Figure 5, A and B). In contrast, *Plp*-Cre-GEdit, *Plp*-Cre-MiniG, and *Plp*-cKI-*Trpa1^mut^* mice developed mechanical allodynia beginning at 24 hours of food deprivation (Supplemental Figure 5A and Figure 5C). Conversely, *Adv*-Cre-GEdit, *Adv*-Cre-MiniG, and *Adv*-cKI-*Trpa1^mut^* mice did not exhibit paw mechanical allodynia following food deprivation (Supplemental Figure 5B and Figure 5D). We next found that the exposure to cold temperature (4 °C) for 15 hours did not induce mechanical allodynia in control mice when assessed up to 4 hours after the exposure (Figure 5, E and F). In contrast, cold temperature induced time-dependent mechanical allodynia in *Plp*-Cre-GEdit, *Plp*-Cre-MiniG, and *Plp*-cKI-*Trpa1^mut^* mice but not in *Adv*-Cre-GEdit, *Adv*-Cre-MiniG, or *Adv*-cKI-*Trpa1^mut^* mice (Supplemental Figure 5, C and D, and Figure 5, G and H). Finally, following a single 2-hour restraint stress, *Plp*-Cre-GEdit, *Plp*-Cre-MiniG, and *Plp*-cKI-*Trpa1^mut^*, but not *Adv*-Cre-GEdit, *Adv*-Cre-MiniG, *Adv*-cKI-*Trpa1^mut^* and control mice, developed mechanical allodynia at 24 hours, which persisted up to 48 hours and gradually resolved by 72 hours after restraint (Supplemental Figure 5, E and F, and Figure 5, I–L). Together, these findings underscore the role of SC TRPA1^mut^ as a key modulator of mechanical allodynia triggered by fasting, cold exposure, and restraint stress in the mouse model of FEPS.

### Schwann cell TRPA1^mut^ amplifies oxidative stress to sustain mechanical allodynia.

The development and maintenance of chronic pain have been linked to oxidative stress/ROS levels within the peripheral nervous system across several animal models of pain (60–63). We previously reported that SC TRPA1 drives a feed-forward mechanism that amplifies and sustains oxidative stress–related nociceptive signaling, thereby contributing to the persistence of mechanical allodynia (47). Having disentangled the distinct contribution of neuronal and SC TRPA1 to acute nociception and sustained mechanical allodynia, respectively, we investigated the role of oxidative stress in the development of mechanical allodynia in FEPS mice.

First, we assessed ROS production in TRPA1 and TRPA1^mut^-HEK293T cells following stimulation with hydrogen peroxide (H_2_O_2_), AITC, or 4-HNE. In both TRPA1 or TRPA1^mut^-HEK293T transfected cells, H_2_O_2_, AITC, and 4-HNE elicited a concentration-dependent increase in intracellular H_2_O_2_ levels with markedly higher potency and efficacy on TRPA1^mut^-HEK293T. These effects were reduced in the presence of A967079 (30 μM) (Figure 6, A–C). Previously,` we observed that the feed-forward amplification of H_2_O_2_ production in Schwann cells was mediated by TRPA1-dependent calcium influx, which activates NADPH oxidase 1 (NOX1) (Supplemental Figure 5G) (64). Accordingly, pharmacological inhibition of NOX1 (ML171, 1 μM) in HEK293T cells (Supplemental Figure 5H) prevented the increase in H_2_O_2_ levels in TRPA1^mut^-HEK293T cells following stimulation with H_2_O_2_, AITC, or 4-HNE (Supplemental Figure 5I). These results were further confirmed in primary murine Schwann cells isolated from *Plp-Cre-GEdit, Plp-Cre-MiniG,* and *Plp-cKI-Trpa1^mut^* mice (Supplemental Figure 5J).

Fasting, cold exposure, and restraint stress conditions evoked mechanical allodynia in *Plp*-Cre-GEdit, *Plp*-Cre-MiniG, and *Plp*-cKI- *Trpa1^mut^* but not in *Adv*-Cre-GEdit, *Adv*-Cre-MiniG, *Adv*-cKI- *Trpa1^mut^* and control mice. The same conditions also induced time-dependent accumulation of 4-HNE within the sciatic nerve of mice carrying the *Trpa1^mut^* mutation in SCs cells, but not in those with a mutation restricted to sensory neurons (Figure 7, A, C, and E, and Supplemental Figure 6, A–C). Notably, the absence of increased 4-HNE staining in the spinal cord white matter of mice carrying the *Trpa1^mut^* mutation in Schwann cells, compared with control mice, exclude a contribution of CNS oligodendrocytes (Supplemental Figure 7, A–C). To further evaluate the demyelination as a potential contributor to the development of mechanical allodynia in mice expressing TRPA1^mut^ in Schwann cells, we assessed the levels of the myelin protein MBP. None of the stress conditions tested altered MBP expression compared with control mice. (Supplemental Figure 7, D–F). To confirm the role of oxidative stress in FEPS mice, we also tested the effect of the antioxidant phenyl-N-tert-butylnitrone (PBN) and the NOX1 inhibitor (ML171) in vivo. Repeated administration of PBN and ML171 reversed mechanical allodynia in *Plp*-cKI-*Trpa1^mut^*, *Plp*-Cre-MiniG, and *Plp*-Cre-GEdit mice following fasting, cold exposure, or restraint stress (Figure 7, B, D, and F, and Supplemental Figure 7, G–L). We speculate that the mutated TRPA1 in Schwann cells detected subthreshold levels of oxidative stress in FEPS mice subjected to different stress conditions. Subsequent activation of TRPA1^mut^ in Schwann cells triggered a feed-forward mechanism that amplifies and sustains mechanical allodynia.

### Schwann cell and neuronal TRPA1^mut^ synergically contribute to mechanical allodynia.

To investigate the potential synergistic interaction between TRPA1^mut^ expressed in sensory neurons and SCs in the development of acute nociception and mechanical allodynia, we generated mice carrying TRPA1^mut^ in both cell populations. *Adv-Cre* mice first received an AAV2/9n vector carrying a Cre-dependent, *loxP*-flanked SaCas9 cassette, thereby restricting SaCas9 expression and genome editing to Cre-expressing primary sensory neurons. The same mice subsequently received an AAVrh10 vector carrying a non-Cre-dependent SaCas9 cassette under the control of the SC-selective *Mpz* promoter, thus targeting genome editing to SCs (Supplemental Figure 8A). Following stimulation with a subthreshold dose of either AITC or 4-HNE, double-mutant mice (*Adv*-Cre-GEdit-double-*Trpa1^mut^*) exhibited enhanced acute nociceptive responses compared with control mice and mice carrying the mutation exclusively in SCs (*Adv*-Cre-GEdit-*Mpz)* (Supplemental Figure 8B), thus recapitulating the phenotypes observed in mice carrying the mutation in sensory neurons (*Adv*-Cre-GEdit). Notably, although in double-mutant mice the magnitude of mechanical allodynia was comparable to that observed in mice carrying the mutation exclusively in SCs (*Adv*-Cre-GEdit-*Mpz*) its duration was prolonged (Supplemental Figure 8C). A prolonged time course in mechanical allodynia was also observed in double-mutant mice following fasting, cold exposure, or restraint stress (Supplemental Figure 8, D–F). We speculate that this sustained hypersensitivity may reflect a feed-forward interaction between SCs and nociceptors, whereby the enhanced production of ROS and RCS by TRPA1^mut^ SCs further activates hypersensitive neuronal TRPA1^mut^, thereby maintaining mechanical allodynia over time.

## Discussion

Here, we provide evidence that, in our mouse models of FEPS, the gain-of-function mutation of the TRPA1 channel exerts a strikingly cell-specific role in pain modulation. By studying TRPA1^mut^ in Schwann cells and neurons, we demonstrate that the mutation differentially regulates acute nociception and mechanical allodynia triggered by acute physiological and environmental challenges, including fasting, cold exposure, and restraint stress. Specifically, TRPA1^mut^ expression in primary sensory neurons enhances the perception of acute painful stimuli, whereas its expression in Schwann cells drives the development and persistence of mechanical allodynia. These findings support a functional dichotomy in TRPA1 signaling and identify Schwann cells as a key cellular substrate through which TRPA1 gain-of-function mutations can promote chronic pain.

Pain research has traditionally focused on neurons as the primary mediators of nociceptive transmission. However, increasing evidence indicates the critical role of nonneuronal cells in the initiation and maintenance of pain states. Our findings do not contradict the dogma that neurons are the final effectors of pain signals; rather, upon injury or insult, nonneuronal cells release neuromodulatory substances in proximity to nociceptors, thereby sustaining pain (65). Following nerve injury, immune cells can infiltrate the peripheral nervous system, releasing proalgesic factors such as leukocyte elastase from T lymphocytes or tumor necrosis factor, interleukin-1β, and ROS from monocytes/macrophages (66–68). In parallel, nonimmune resident cells such as keratinocytes and satellite glial cells secrete neuroactive molecules, such as prostaglandin E2, ATP, nerve growth factor, and matrix metalloproteinases, which directly modulate nociceptor excitability (69–72).

We have previously shown that Schwann cells, the primary glial cells of the peripheral nervous system, acquire a proalgesic phenotype in multiple mouse models of pain, releasing ROS that sensitize nearby nociceptors (39, 40, 42, 44, 45, 47, 73). In this study, we extend the role of Schwann cells in a genetically driven pain syndrome, demonstrating that TRPA1 gain-of-function signaling in these cells is sufficient to maintain persistent mechanical allodynia independently of neuronal TRPA1^mut^ expression. To dissect the cell-specific contribution of TRPA1^mut^ in FEPS, we generated 3 complementary transgenic mouse models of FEPS carrying the TRPA1^mut^ mutation in Schwann cells and primary sensory neurons. We found that i.pl. administration of subthreshold doses of 2 different selective TRPA1 agonists (AITC and 4-HNE) elicited a robust acute nociceptive response in mice expressing the TRPA1^mut^ in primary sensory neurons that was ineffective in WT mice. In contrast, mice expressing TRPA1^mut^ in Schwann cells developed pronounced mechanical allodynia, a phenotype absent in both WT mice and in animals expressing TRPA1^mut^ in primary sensory neurons.

Individuals suffering from chronic pain frequently avoid conditions or activities that could worsen their symptoms. It is widely reported that prolonged or excessive stress, including sleep disturbances, can induce dysfunction of cortisol activity, leading to widespread inflammation and enhanced pain sensitivity (74,75). Similarly, barometric changes trigger imbalances in brain chemicals, including serotonin, which facilitate migraine attacks (76). Acute physical activity and exercise induce phosphorylation of the NMDA receptor in peripheral nerves, a mechanism that can promote pain sensitization in chronic pain conditions (77). Importantly, patients with familial FEPS report that episodes of severe pain are often precipitated by physiological or environmental stressors, including fasting, cold exposure, or psychological stress (27). In line with these observations, we modeled food deprivation, cold exposure, and restraint-induced stress in mice. Under these subthreshold discomfort conditions, mice expressing TRPA1^mut^ selectively in primary sensory neurons did not develop mechanical allodynia, similar to WT controls. In sharp contrast, mice expressing TRPA1^mut^ in Schwann cells displayed robust pain behaviors under the same conditions, indicating that Schwann cell–mediated TRPA1 signaling is sufficient to convert mild environmental or metabolic stressors into prolonged painful responses, highlighting a critical role for nonneuronal TRPA1 activity in FEPS pathogenesis.

The contribution of ROS in chronic pain is widely recognized (78–82). We have previously demonstrated that, in Schwann cells, TRPA1 plays a critical role in ROS production and amplification, a process tightly correlated to the onset and persistence of chronic pain. Several proalgesic mediators, such as CGRP in periorbital allodynia, CSF-1 in primary tumor, IGF-1 in bone metastatic pain, and C5a in endometriosis pain, activate intracellular pathways in Schwann cells that ultimately lead to TRPA1 activation, excessive ROS generation, and paracrine sensitization of primary sensory neurons (40, 44, 48). Here, we show that the TRPA1^mut^ channel was activated by subthreshold doses of ROS, leading to TRPA1-dependent H_2_O_2_ release, an effect not observed in WT TRPA1. Moreover, in vivo models of food deprivation, cold exposure, and constraint-induced stress resulted in ROS accumulation in the peripheral nerves of mice with Schwann cell TRPA1^mut^, but not in WT mice or in animals carrying the TRPA1 mutation in primary sensory neurons. We speculate that these mild stress conditions activated intracellular pathways in Schwann cells that remain below the activation threshold of WT TRPA1 but are sufficient to engage TRPA1^mut^, thereby initiating a feed-forward mechanism of ROS production and sustained nociceptor sensitization. The accumulation of 4-HNE observed under these conditions may further support paracrine signaling between myelinating Schwann cells and unmyelinated nociceptors, helping to overcome the potential spatial attenuation of ROS, which could otherwise be limited by structural features such as Remak bundles. Although ROS, and in particular H_2_O_2_, can diffuse and remain active over distances of up to approximately 150 μm (64, 83, 84), and the physical distance between a myelinating Schwann cell and axons within Remak bundles is less than 1 μm (85), lipid peroxidation products such as 4-HNE are more stable and can diffuse across cellular compartments and into the extracellular space, acting as secondary messengers far from the site of origin (86). In this context, we cannot exclude that the Schwann cell TRPA1–dependent ROS production may secondarily target additional redox-sensitive pathways involved in nociceptive signaling, thereby amplifying pain transmission, including TRP vanilloid 1 (TRPV1), TRP melastatin 2 (TRPM2), acid-sensing ion channel subunit 1a (ASIC1a), P2X7 receptors, or the NOD-, LRR- and pyrin domain–containing protein 3 (NLRP3) inflammasome (87–91). Therefore, Schwann cell TRPA1 may act as an upstream redox amplifier that converts an initial oxidative signal into a broader pronociceptive and neuroinflammatory response.

Importantly, the TRPA1^mut^ gain-of-function mutation results in enhanced channel activity, such that even a limited number of cells expressing the mutant channel can produce a substantial functional impact. In Schwann cells, this effect is likely further amplified by noncell-autonomous mechanisms, including feed-forward oxidative signaling and paracrine sensitization of neighboring nociceptors. Together, these considerations provide a plausible explanation for the robust behavioral phenotype observe,d despite partial editing/replacement efficiency. The double-mutant model further supports this concept. Mice expressing TRPA1^mut^ in both Schwann cells and sensory neurons did not exhibit an increase in mechanical allodynia than mice carrying the mutation selectively in Schwann cells, but rather a markedly prolonged duration of hypersensitivity. This finding suggests that the enhanced sensitization characteristic of FEPS do not simply reflect increased pain intensity but instead result from the cooperative interaction between hypersensitive Schwann cells and nociceptors, which sustains nociceptive signaling over time.

In conclusion, our study dissects the contributions of TRPA1 in Schwann cells and primary sensory neurons to mechanical allodynia and acute pain in FEPS mice. It provides complementary transgenic models to investigate the distinct roles of neuronal and nonneuronal TRPA1 signaling in chronic pain disorders.

### Limitations of the Study.

One limitation of this study is that, whereas patients with FEPS predominantly report upper-body pain (27), mechanical hypersensitivity in mice was assessed on plantar surface of the hind paw. This difference likely reflects the use of standardized, robust, and sensitive behavioral readout in rodents, rather than a direct anatomical correspondence with the human phenotype. Nevertheless, we speculate that TRPA1-dependent mechanisms may generate a generalized state of enhanced nociceptive sensitivity, which in humans preferentially manifests in upper-body regions, possibly reflecting species-specific anatomical, cellular, and molecular features of sensory neurobiology.

A second limitation is that the present study introduced the FEPS-associated mutation into the endogenous murine Trpa1 sequence, rather than comparing humanized knock-in mice expressing hTRPA1-WT and hTRPA1-N855S. This strategy was chosen to preserve the native murine genomic, transcriptional, and cellular context of Trpa1 expression and to enable cell type–specific dissection of mutant TRPA1 function in sensory neurons and Schwann cells. Nevertheless, given the known species-dependent differences in TRPA1 pharmacology and cold sensitivity, humanized TRPA1 knock-in models would represent a valuable complementary approach to further assess the translational relevance of human TRPA1-targeted interventions.

A further limitation is that, although this study provides insights into the cellular mechanisms underlying pain in patients with FEPS, it does not directly offer a therapeutic strategy capable of providing long-term relief from chronic pain. Nonetheless, the animal model established here represents a valuable platform for the development of targeted biotechnological approaches, such as selective silencing of TRPA1 expression in Schwann cells or gene therapy strategies to modify the genetic sequence, aimed at reducing cellular hypersensitivity to discomfort in patients with FEPS.

## Methods

### Sex as a biological variable.

Male and female mice were included in all experiments as mixed-sex cohorts, reflecting the sex distribution of individuals carrying the mutation in the original pedigree. To evaluate sex as a biological variable, data were initially analyzed using mixed-effects models with sex, genotype, and treatment as fixed effects, including all interaction terms (see Supplemental Data). As no significant sex-dependent differences or sex-related interactions were identified, data from male and female mice were pooled for final analyses and presentation.

A detailed description of experimental procedures, plasmid and primer sequences (Supplemental Table 1), viral-vector production, and analytical workflows are provided in the Supplemental Materials.

### Mice.

Male and female mice were used throughout (25–30 g, 7–9 weeks). The following mouse strain was used: C57BL/6J mice (Charles River, RRID: IMSR_JAX:000664). The C57BL/6N-*Trpa1^tm1.1Ics^* (*Trp1a^cKI^*) mutant mouse line was generated at the Institut Clinique de la Souris - PHENOMIN (http://www.phenomin.fr*)*. To produce the conditional N855S knock-in Trpa1 mouse model, a targeting plasmid was engineered and electroporated into C57BL/6N embryonic stem (ES) cells. The construct contained two distinct pairs of recombination sites, one pair of WT *loxP* sites and one pair of *lox511* sites, each arranged in a head-to-head orientation. The targeting vector included the murine exon 22 sequence together with its flanking intronic regions cloned in the sense orientation, and a modified degenerated human exon 22 sequence harboring the N855S mutation along with its corresponding human intronic flanking regions cloned in the antisense orientation (Figure 4A). Degenerated human exon 22 was generated by exploiting the degeneracy of the genetic code. Multiple synonymous substitutions were introduced into the DNA sequence while preserving the encoded amino acid sequence. Both *loxP* and *lox511* sites were positioned in an inverted orientation and are recognized by Cre recombinase; however, *lox511* sites only recombine efficiently with identical *lox511* sites and not with *loxP* sites. Upon Cre-mediated recombination, an initial inversion event occurs at either the *loxP* or *lox511* sites, which are present in opposite orientations, resulting in the formation of two identical recombination sites (either *loxP*–*loxP* or *lox511*–*lox511*) in the same orientation. Subsequent Cre activity leads to excision of the intervening DNA sequence between the recombination sites. This recombination strategy ultimately yields an allele containing a single *loxP* site and a single *lox511* site, thereby preventing further inversion events. Following recombination, the endogenous promoter drives expression of the mutant *Trpa1* allele in place of the WT *Trpa1* gene. To introduce *Trpa1* N855S point mutation in Schwann cells or primary sensory neurons, homozygous *Trpa1^cKI^* were crossed with hemizygous *Plp-Cre^ERT^* mice or hemizygous *Adv-Cre* mice, respectively, as for the *Trpa1^fl/fl^* mice, homozygous *Trpa1^cKI/cKI^* mice (*Plp-Cre^ERT^; Trpa1^cKI/cKI^ and Plp-Cre^ERT–^; Trpa1^cKI/cKI^*, respectively) were subjected to i.p. injection of 4-hydroxytamoxifen (4-OHT, 1 mg/100 μL in corn oil once a day consecutively for 3 days) (47). To generate mice in which the *Trpa1* gene was conditionally silenced in Schwann cells, homozygous 129S-Trpa1^tm2Kykw/J^ (*fl* Trpa1, *Trpa1^fl/fl^*, RRID:IMSR_JAX: 008649 Jackson Laboratory) were crossed with hemizygous B6.Cg-Tg(Plp1-CreERT)3Pop/J mice (*Plp-Cre^ERT^*, RRID: IMSR_JAX:005975 Jackson Laboratory) expressing a tamoxifen-inducible Cre in Schwann cells (Plp1, proteolipid protein myelin 1). The progeny (*Plp-Cre^ERT^;Trpa1^fl/fl^*) was genotyped using PCR for *Trpa1* and *Plp-Cre^ERT^*. Mice that were negative for *Plp-Cre^ERT^* (*Plp-Cre^ERT–^;Trpa1^fl/fl^*) were used as control. Both positive and negative mice for *Cre^ERT^* and homozygous *fl* Trpa1 (*Plp-Trpa1* and control respectively) were treated with 4-OHT, 1 mg/100 μL, i.p., once a day consecutively for 3 days. To selectively delete *Trpa1* in primary sensory neurons, Trpa1^fl/fl^ mice were crossed with hemizygous Advillin-Cre mice (Adv-Cre) (92). Mice positive or negative for Cre and homozygous for *fl* Trpa1 (*Adv-Trpa1;Trpa1^fl/fl^* and control respectively) were used. The successful Cre-driven deletion of TRPA1 mRNA was confirmed using reverse transcription quantitative real-time PCR (RT-qPCR).

*Plp-Cre^ERT^, Adv-Cre, Plp-Cre^ERT^;Trpa1^fl/fl^, Adv-Trpa1;Trpa1^fl/fl^* and control mice were infected with an i.v. or intrathecal (i.th.) (10 μL, 1 × 10^12^ vg/ml) injection of different AAVs and animals were used 3 weeks after AAVs infection.

The group size of *n* = 8 animals for behavioral experiments was determined by sample size estimation using G*Power (v3.1;(93)) to detect size effect in a post-hoc test with type 1 and 2 error rates of 5 and 20%, respectively. Mice were allocated to vehicle or treatment groups using a randomization procedure (http://www.randomizer.org/). The assessors were blinded to the identity of the animals (allocation to treatment groups). None of the animals were excluded from the study. Behavioral studies followed Animal Research: Reporting of In Vivo Experiments (ARRIVE) (94). Animals were euthanized with inhaled CO_2_ plus 10-50% O_2_. Mice were housed in a temperature (20 ± 2 °C) and humidity (50 ± 10%) controlled *vivarium* (12 h dark/light cycle, free access to food and water, five animals per cage). At least 1 h before behavioral experiments, mice were acclimatized to the experimental room and behavior was evaluated between 9:00 am and 5:00 pm. All the procedures were conducted following the current guidelines for laboratory animal care and the ethical guidelines for investigations of experimental pain in conscious animals set by the International Association for the Study of Pain (95). Animals were anesthetized with a mixture of ketamine and xylazine (90 mg/kg and 3 mg/kg, respectively, i.p.).

### Treatments and behavioral testing.

Pharmacological agents were administered locally or systemically at the doses and times specified in the Supplementary Methods. Acute nociception was quantified from paw licking and lifting after intraplantar challenge. Mechanical sensitivity was measured using von Frey filaments and the up-and-down method. The effects of fasting, restraint stress, and cold exposure were assessed using standardized paradigms.

### Cell culture and molecular studies.

HEK293T and AAVpro 293T cells, commercial primary human Schwann cells, mouse Schwann cells isolated from sciatic nerves, and mouse dorsal root ganglion neurons were cultured under the conditions detailed in the Supplementary Methods. Gene expression was measured by RT-qPCR. WT and mutant TRPA1 constructs and Cre-dependent AAV vectors were generated and validated using the listed plasmids, primers, and sequencing procedures.

### AAV production and functional assays.

Recombinant AAVs were produced in AAVpro 293T cells, purified by iodixanol-gradient ultracentrifugation, concentrated, and titrated before use. TRPA1 function was evaluated by whole-cell patch-clamp electrophysiology. Intracellular hydrogen peroxide was monitored in live cells using the genetically encoded HyPer7.2 probe. Tissue and cellular expression were assessed by immunofluorescence with the antibodies and imaging parameters reported in the Supplementary Methods.

### RNA sequencing and bioinformatics.

RNA isolated from sciatic nerves underwent library preparation and sequencing. Reads were quality filtered, aligned to the reference genome, quantified, and analyzed for differential expression and pathway enrichment using the software, versions, parameters, and statistical thresholds specified in the Supplemental Methods.

### Statistics.

Results are expressed as the mean ± SEM. Two groups were compared using 2-tailed Student’s *t* test. Multiple groups were analyzed by 1-way ANOVA followed by Bonferroni correction, and repeated-measures behavioral data by 2-way ANOVA with the indicated post hoc comparisons. Statistical analyses were performed on raw data using GraphPad Prism 8 (GraphPad Software Inc.). *P* values less than 0.05 (P < 0.05) were considered significant. EC_50_ values were determined from nonlinear regression models using GraphPad Prism 8. Exact tests and sample sizes are reported in the figure legends.

### Study approval.

Animal experiments were carried out in accordance with European Union (EU) guidelines for animal care procedures and the Italian legislation (DLgs 26/2014) application of the EU Directive 2010/63/EU. The study was approved by the National Committee for the Protection of Animals used for Scientific Purposes of the Italian Ministry of Health (research permits # 245/2023-PR).

### Data availability.

All materials used in this study are available to any researcher for purposes of reproducing or extending the analyses. Values for all data points shown in graphs and values behind any reported means are reported in the Supporting Data Values file. Fastq sequencing data generated in this study are available in the Sequence Read Archive (SRA) under BioProject accession number PRJNA1424206.

## Manuscript information

### Author contributions.

Conceptualization, E Coppi, M Montini, KYK, MB, RL, RN, FDL; data curation, M Marini, MC, E Colzi, LB, LL, IS, RN, FDL; formal analysis, M Marini, MC, E Coppi, AP, GDS, EB, LT, VA, GF; funding acquisition, FDL; investigation, M Marini, MC, E Colzi, M Montini, LB, LL, IS, AP, GDS, EB, LT, VA, GF, EDNM, MB, RL, RN, FDL; methodology, M Marini, MB, E Coppi, M Montini, GDS, EC, MC, RL; project administration, RN, FDL; resources, RN, FDL; supervision; RN, FDL; writing-original draft, M Marini, MC, E Coppi, M Montini, KYK, MB, RL, RN, FDL; writing – review & editing, Marini, MC, E Coppi, KYK, MCB, RN, FDL Co–first author position was determined by the order in which those authors joined the project.

### AI disclosure.

The authors declare that no AI tools were used in the original research for or writing of this article.

### Conflict of interest.

The authors have declared that no conflict of interest exists.

### Funding support.

This work was sponsored by:

- Fondazione Telethon (Grant no GMR22T1070) (to FDL).

## Supplementary Material

Supplemental data

Supporting data values

## Figures and Tables

**Figure 1:**
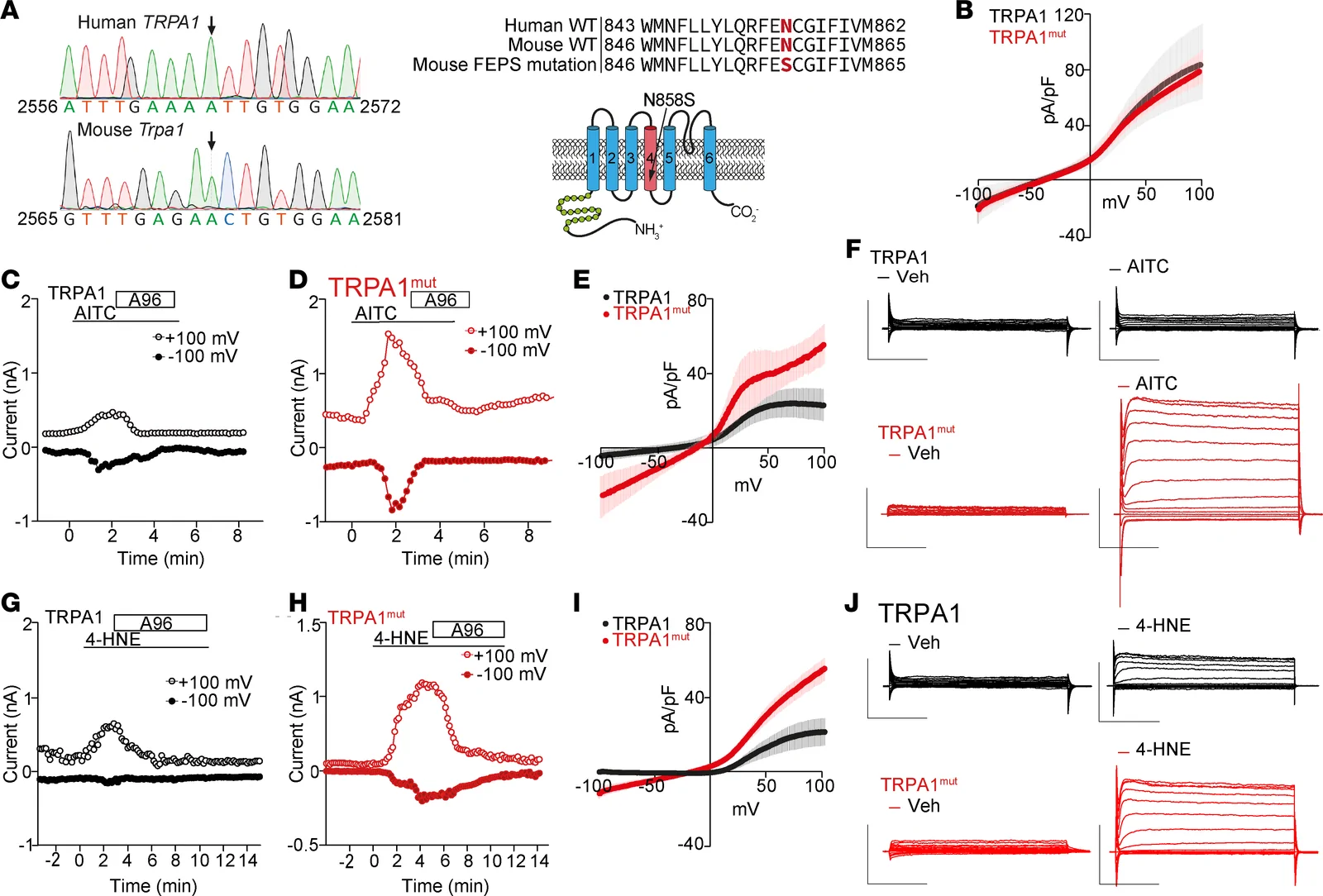
HEK293 cells transfected with the mutant mTRPA1^mut^ channel present enhanced currents upon stimulation with exogenous or endogenous agonists. (**A**) Sanger sequence chromatogram showing human *TRPA1* and mouse *Trpa1*. Amino acid sequences are conserved between WT human and mouse genes. Schematic representation of FEPS mutation (N858S) in transmembrane domain 4 of TRPA1 channel. (**B**) Voltage-dependent currents activated a depolarizing voltage ramp protocol (from –100 to +100 mV; 800 ms) in HEK293 cells transfected with the mouse TRPA1 channel (TRPA1, *n* = 12) or its mutated counterpart (TRPA1^mut^, *n* = 15). (**C** and **D**) Time course of current amplitude evoked by the voltage ramp at + 100 mV or –100 mV in a TRPA1- (**C**) or TRPA1^mut^- (**D**) HEK293T transfected cells after AITC (100 μM) also in the presence of A967079 (A96) (100 μM). (**E**) AITC-activated currents measured in TRPA1- (*n* = 6) or in TRPA1^mut^- (*n* = 6) HEK293T transfected cells by subtraction of the control ramp from that recorded in AITC. (**F**) Original current traces evoked by a depolarizing voltage-step protocol (from –140 to +140 mV; 20 mV increment; 300 ms steps) recorded before or after the application of AITC in a TRPA1 or TRPA1^mut^- HEK293T transfected cell (**G** and **H**) Time course of current amplitude evoked by the voltage ramp at + 100 mV or –100 mV in a TRPA1- (**C**) or TRPA1^mut^- (**D**) transfected HEK293 cells after 4-HNE (100 μM) and in the presence of A96 (100 μM). (**I**) 4-HNE–activated currents measured in TRPA1- (*n* = 6) or in TRPA1^mut^- (*n* = 9) HEK293T transfected cells. (**J**) Original current traces evoked by a depolarizing voltage-step protocol recorded before or after the application of 4-HNE in a TRPA1- or TRPA1^mut^-HEK293T transfected cells. Data are mean ± SEM. Veh, vehicle.

**Figure 2:**
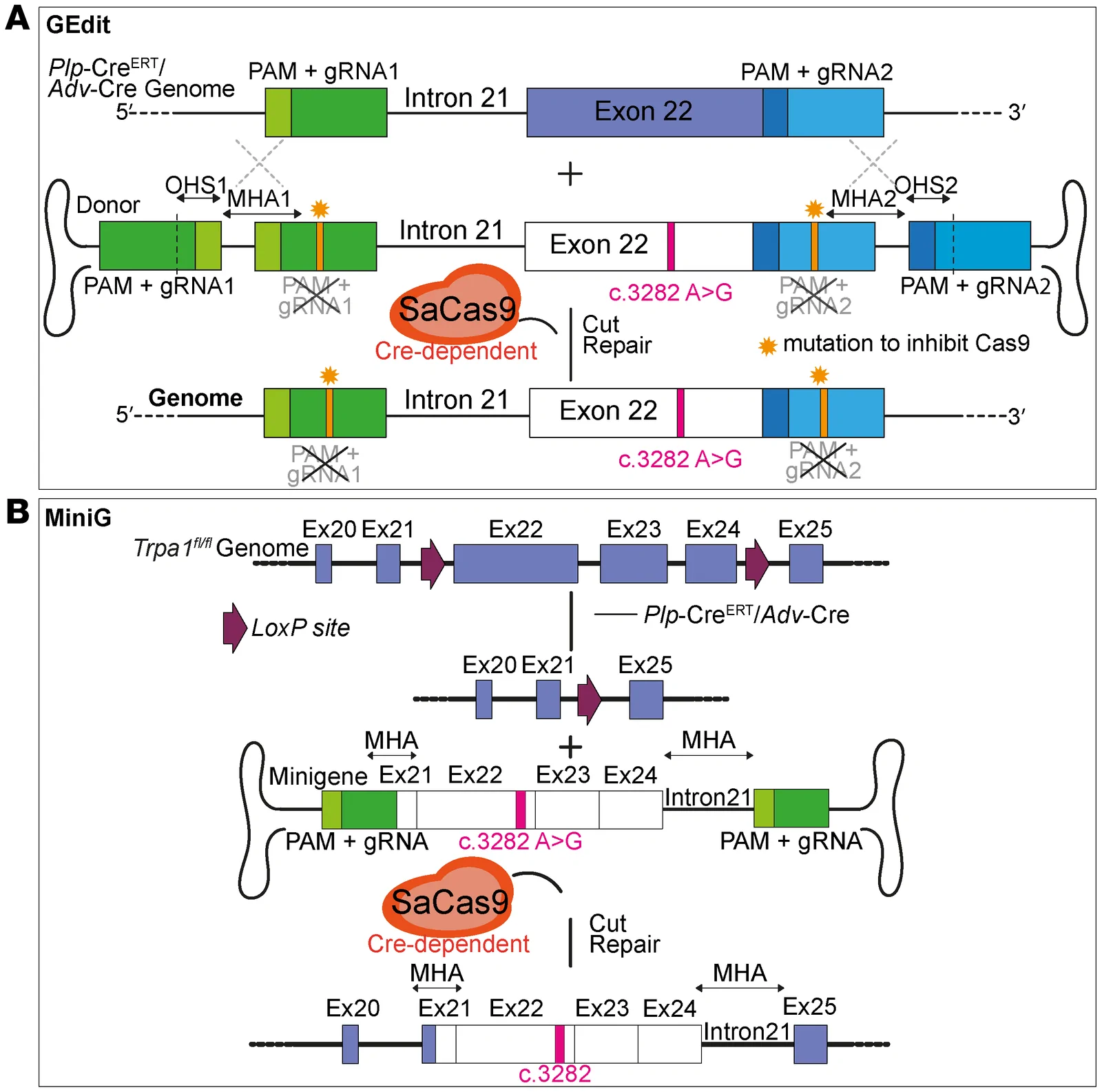
Generation of mouse model with c.3282 A>G mutation on exon 22 of Trpa1 gene. (**A**) Schematic illustration of genome editing approach (GEdit) based on MMEJ mutation substitution. *Plp*-Cre^ERT^ or *Adv*-Cre mice genome and donor DNA with c.3282 A>G mutation on exon 22 carried by AAV were excised at the flanking gRNA target sites (gRNA1 and gRNA2) by SaCas9. Donor DNA with c.3282 A>G is inserted into the mouse genome using microhomology arms (MHA) inducing the FEPS mutation. Single point mutations in gRNA1 and gRNA2 were designed to prevent SaCas9 repeated cleavage after successful replacement. (**B**) Schematic representation of minigene approach (MiniG) based on Cre-*Trpa1^fl/fl^* mice where exons 22–24 of *Trpa1* were flanked by *LoxP* sites. In presence of Cre, exons 22–24 of WT *Trpa1* were excised. Minigene with the exons 22-24 was excised at the flanking gRNA target sites by SaCas9 and inserted into the mouse genome, inducing c.3282 A>G mutation. PAM, protospaces adjacent motif; MHA, micro-homology arm.

**Figure 3:**
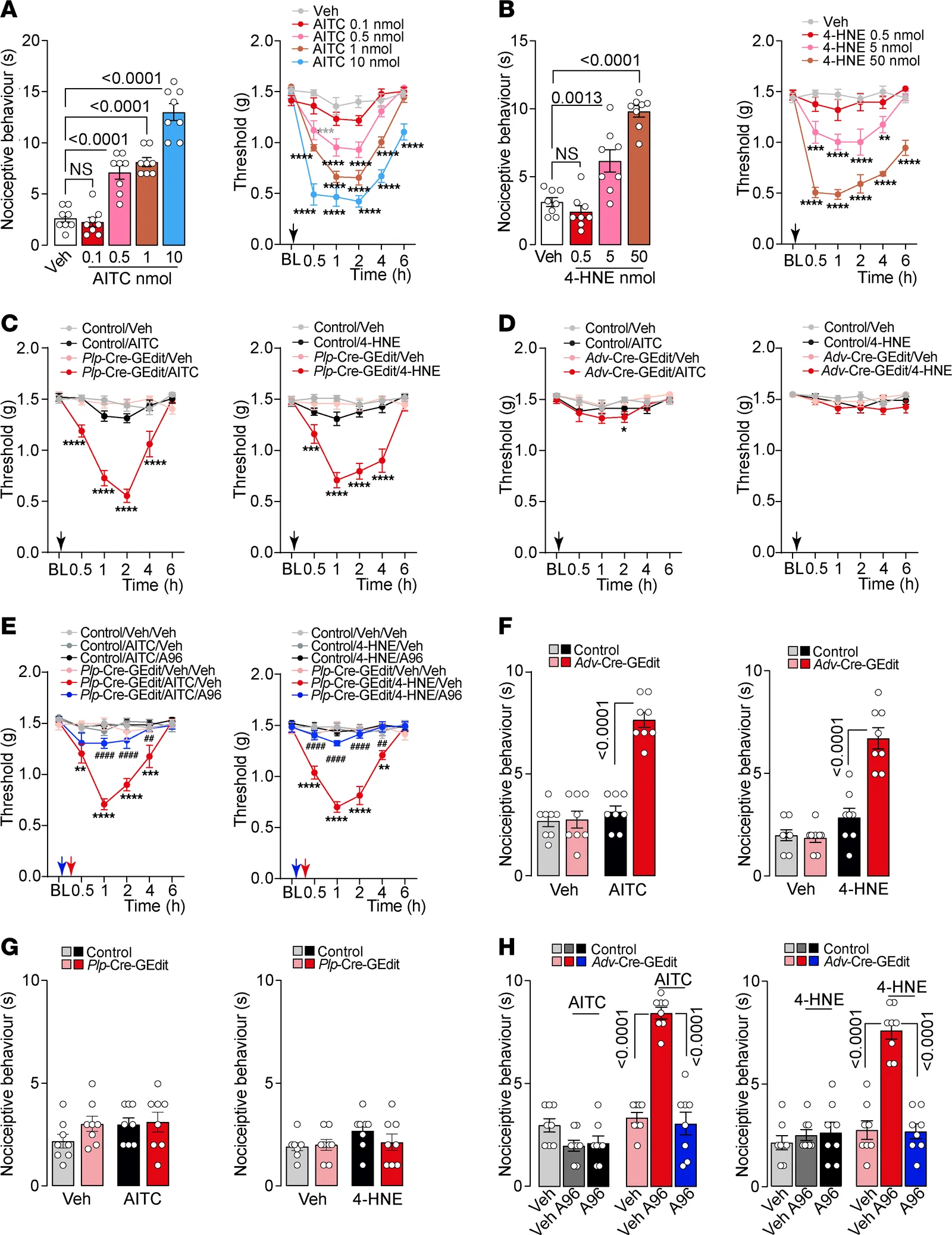
Mutant TRPA1 in Schwann cells and primary sensory neurons independently mediates mechanical allodynia and acute nociception. (**A** and **B**) Dose-dependent, acute nociception and dose- and time-dependent hind paw mechanical allodynia after intraplantar (i.pl./10 ml) injection of AITC and 4-HNE or vehicle (Veh) in C57BL/6J mice (B6). (**C** and **D**) Mechanical allodynia after i.pl. AITC (0.1 nmol), 4-HNE (0.5 nmol), or Veh in *Plp-*Cre-GEdit, *Adv-*Cre-GEdit or control mice. (**E**) Mechanical allodynia after i.pl. AITC (0.1 nmol), 4-HNE (0.5 nmol), or Veh in *Plp-*Cre-GEdit mice pretreated with A967079 (A96, 100 mg/kg, i.p.) or Veh. (**F** and **G**) Acute nociceptive response after i.pl. AITC (0.1 nmol), 4-HNE (0.5 nmol), or Veh in *Adv-*Cre-GEdit, *Plp-*Cre-GEdit, or control mice. (**H**) Acute nociception after i.pl. AITC (0.1 nmol), 4-HNE (0.5 nmol) or Veh in *Adv-*Cre-GEdit mice pretreated with A96 (100 mg/kg, i.p.). Data are mean ± SEM. (*n* = 8 mice/group). Arrow indicates time of compound administration. 1-way (**A** and **B** left, **F**–**H**) and 2-way ANOVA (**A** and **B** right, **C**–**E**) Bonferroni correction. *P < 0.05, **P < 0.01, ***P < 0.001, ****P < 0.0001 versus Veh or versus Control/Veh or versus Control/Veh/Veh; ^##^*P* < 0.01, ^####^*P* < 0.0001vs *Plp-*Cre-GEdit/HNE/AITC/Veh. Veh, vehicle.

**Figure 4:**
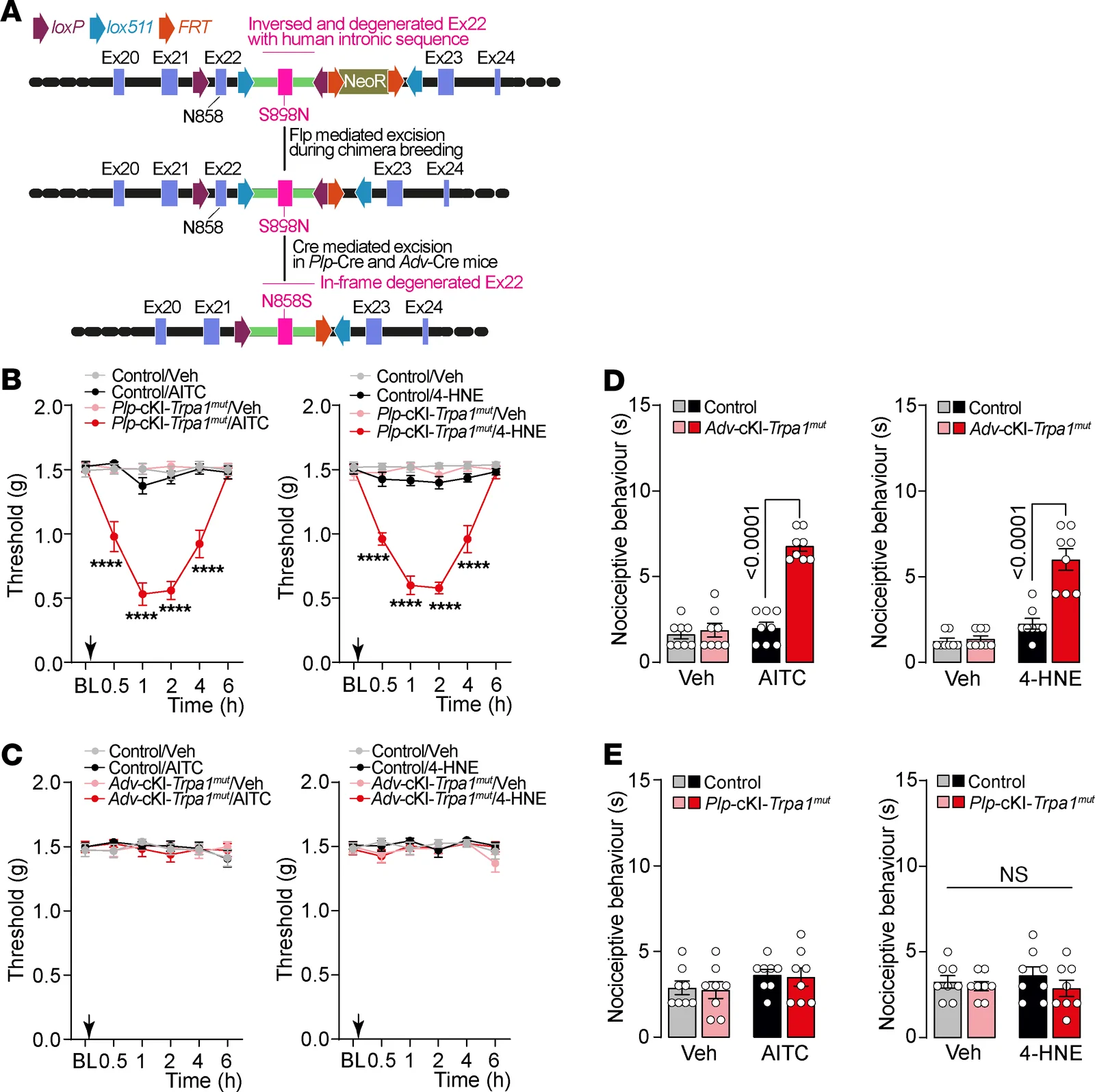
Knock-in FEPS mouse model reveals specific role of mutant TRPA1 in Schwann cells and primary sensory neurons in mechanical allodynia and acute nociception. (**A**) Schematic representation of conditional knock-in FEPS mouse model. (**B** and **C**) Time-dependent mechanical allodynia after intraplantar (i.pl./10 ml) injection of AITC (0.1 nmol), 4-HNE (0.5 nmol) or vehicle (Veh) in *Plp*-cKI-*Trpa1^mut^, Adv*-cKI-*Trpa1^mut^* or Control mice. (**D** and **E**) Acute nociception after i.pl. AITC (0.1 nmol), 4-HNE (0.5 nmol), or Veh in *Adv*-cKI-*Trpa1^mut^, Plp*-cKI-*Trpa1^mut^,* or control mice. Data are mean ± SEM. (*n* = 8 mice/group). Arrow indicates time of compounds administration. 2-way (**B** and **C**) and 1-way (**D** and **E**) ANOVA, Bonferroni correction. ****P < 0.0001 versus Control/Veh.

**Figure 5:**
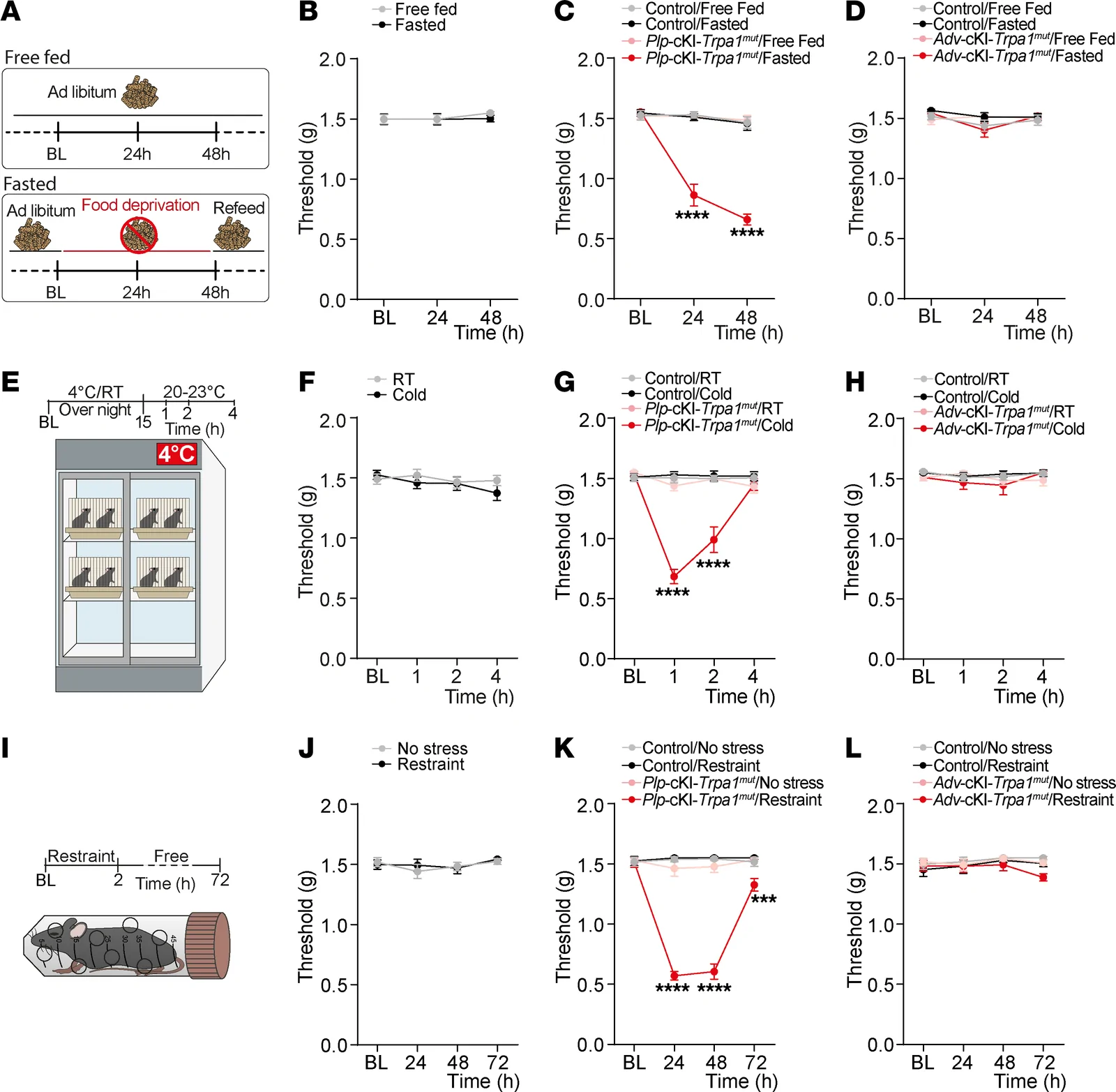
Fasting, cold exposure, and restraint stress induce Schwann cell–dependent mechanical allodynia mediated by mutant TRPA1. (**A**) Schematic representation of free or fasted diet regimen. (**B**–**D**) Time dependent mechanical allodynia in free fed or fasted *Plp*-cKI-*Trpa1^mut^*, *Adv*-cKI-*Trpa1^mut^* or control mice. (**E**) Schematic representation of cold (4 °C) or room temperature (RT) exposure (**F**–**H**) Time dependent mechanical allodynia in *Plp*-cKI-*Trpa1^mut^*, *Adv*-cKI-*Trpa1^mut^*, or Control mice (*n* = 8 mice/group) exposed to cold or RT. (**I**) Schematic representation of restraint stress model. (**J**–**L**) Time-dependent mechanical allodynia in the presence or absence of restraint stress in *Plp*-cKI-*Trpa1^mut^*, *Adv*-cKI-*Trpa1^mut^*, or control mice. Data are mean ± SEM. (*n* = 8 mice/group). 2-way ANOVA (**B**–**D**, **F**–**H**, and **J**–**L**), Bonferroni correction. ****P < 0.0001 versus Control/Fasted, Control/Cold, Control/Restraint.

**Figure 6:**
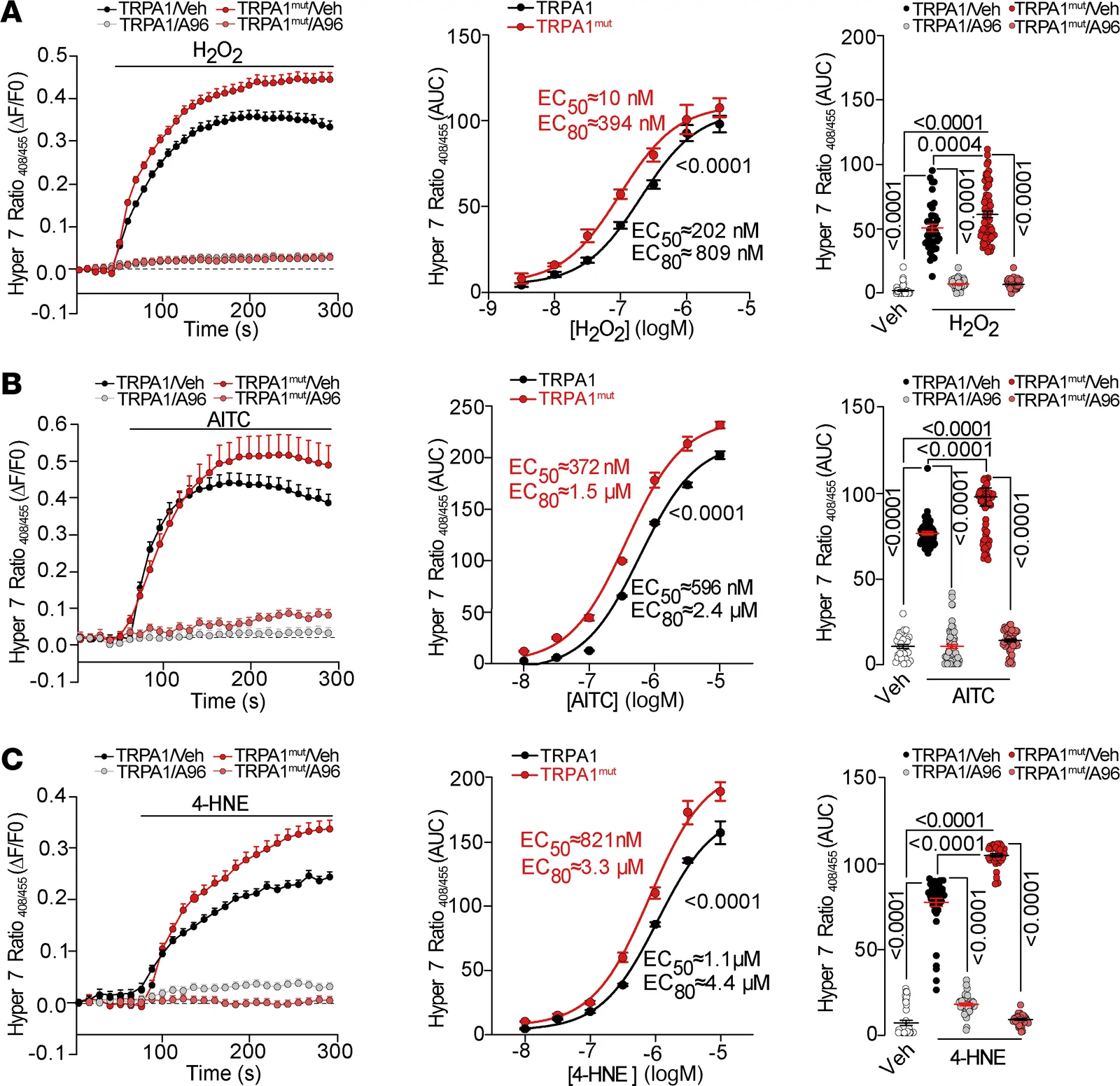
Mutant TRPA1 channel generates oxidative stress. (**A**–**C**) Typical traces, concentration response curve (CRC), and pooled data of HyPer7.2 in HEK293T cells transfected with the mouse TRPA1 channel (TRPA1) or its mutated counterpart (TRPA1^mut^), stimulated with H_2_O_2_ (3 nM-3 μM), AITC (10 nM-10 μM), and 4-HNE (10 nM-10 μM) in the presence of A967079 (A96, 30 μM) or vehicle (Veh, 0.0001% DMSO) (cells number: H_2_O_2_ CRC TRPA1: 3 nM *n* = 56; 10 nM *n* = 46; 30 nM *n* = 57; 100 nM *n* = 66; 300 nM *n* = 6; 1 μM *n* = 66; 3 μM *n* = 65. EC_50_CI = 149–275 nM; EC_80_CI = 598 nM–1.1 μM. H_2_O_2_ CRC TRPA1^mut^: 3 nM *n* = 50; 10 nM *n* = 103; 30 nM *n* = 66; 100 nM *n* = 63; 300 nM *n* = 43; 1 μM *n* = 47; 3 μM *n* = 56. EC_50_CI = 7–144 nM, EC_80_CI = 271–577 nM. H_2_O_2_ cumulative data: Veh *n* = 48; TRPA1/Veh *n* = 42; TRPA1/A96 *n* = 66; TRPA1^mut^ /Veh *n* = 67; TRPA1^mut^ /A96 *n* = 66; AITC CRC TRPA1: 10 nM *n* = 93; 30 nM *n* = 87; 100 nM *n* = 55; 300 nM *n* = 73; 1 μM *n* = 68; 3 μM *n* = 55; 10 μM *n* = 88. EC_50_CI = 549–649 nM, EC_80_CI = 2.2–2.6 μM. AITC CRC TRPA1^mut^: 10 nM *n* = 36; 30 nM *n* = 31; 100 nM *n* = 43; 300 nM *n* = 71; 1 μM *n* = 66; 3 μM *n* = 55; 10 μM *n* = 57. EC_50_CI = 311–444 nM, EC_80_CI = 1.2–1.8 μM. AITC cumulative data: Veh *n* = 31, TRPA1/Veh *n* = 73; TRPA1/A96 *n* = 64; TRPA1^mut^/Veh *n* = 61; TRPA1^mut^ /A96 *n* = 50; 4-HNE CRC TRPA1: 10 nM *n* = 55; 30 nM *n* = 60; 100 nM *n* = 47; 300 nM *n* = 56; 1 μM *n* = 40; 3 μM *n* = 46; 10 μM *n* = 37. EC_50_CI = 926 nM–1.3 μM; EC_80_CI = 3.7–5.3 nM. 4-HNE CRC TRPA1^mut^: 10 nM *n* = 82; 30 nM *n* = 46; 100 nM *n* = 50; 300 nM *n* = 35; 1 μM *n* = 23; 3 μM *n* = 61; 10 μM *n* = 66. EC_50_CI = 617 nM–1.2 μM; EC_80_CI = 2.5–4.4 μM. 4-HNE cumulative data: Veh *n* = 39; TRPA1/Veh *n* = 44; TRPA1/A96 *n* = 48; TRPA1^mut^/Veh *n* = 38; TRPA1^mut^/A96 *n* = 46). Data are mean ± SEM. 1-way (**A**–**C**) ANOVA, Bonferroni correction.

**Figure 7:**
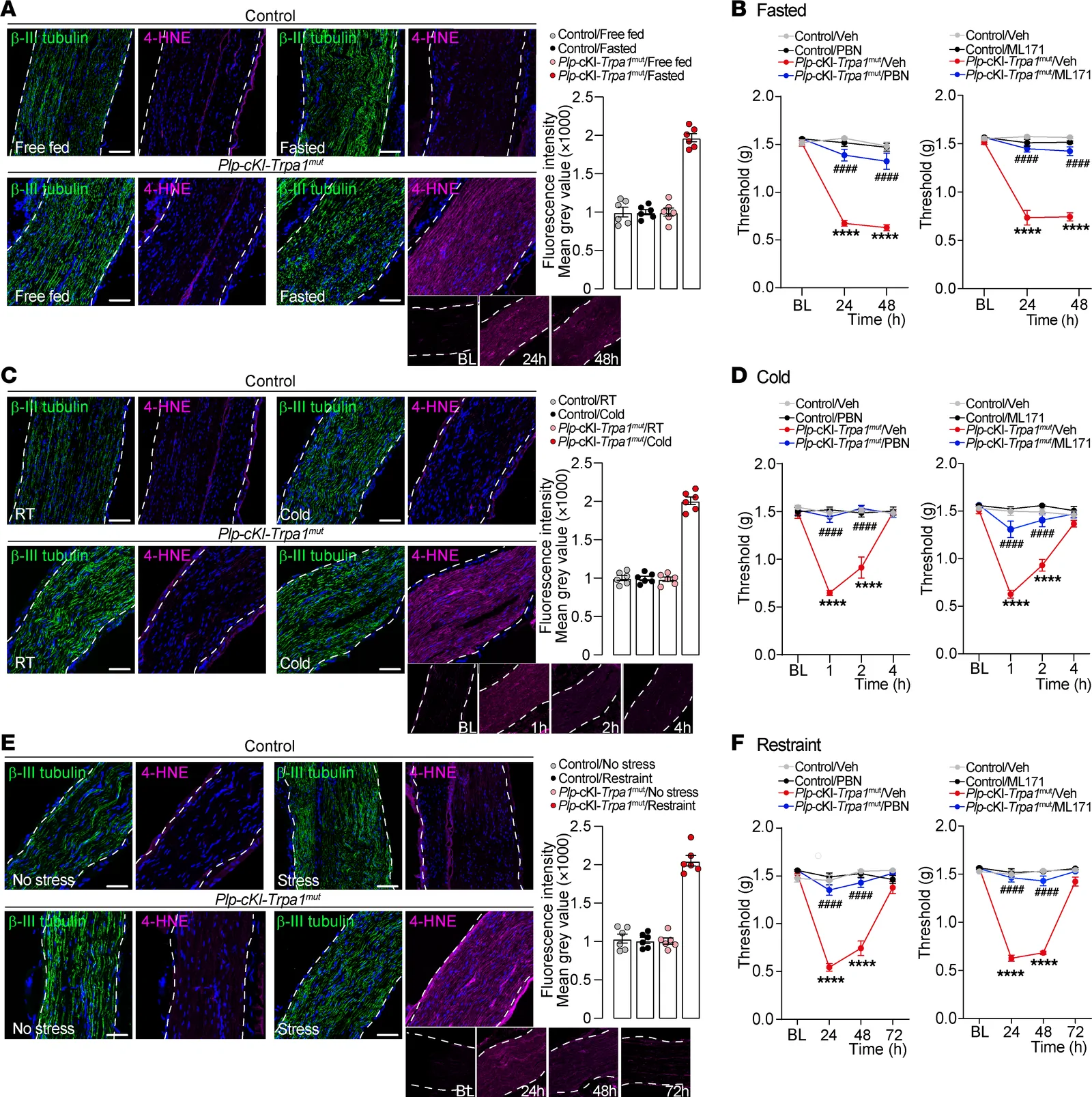
Oxidative stress generated by mutant TRPA1 channel maintains mechanical allodynia. (**A**, **C**, and **E**) Representative images and cumulative data of 4-HNE content within the sciatic nerve of *Plp*-cKI-*Trpa1^mut^*or control mice (**A**) free fed or fasted, (**C**) exposed to room temperature (RT) or cold or (**E**) in the presence or absence of restraint stress. (*n* = 6, scale bar: 50 μm). The main representative images show 4-HNE levels at (**A**) 24 hours after fasting, (**C**)1 hour after cold stress, and (**E**) 24 hours after restraint stress model. Insets show the time course of 4-HNE accumulation of each stress model. (**B**, **D**, and **F**) Time dependent mechanical allodynia following repeated administration of phenyl-N-tert-butylnitrone (PBN, 100 mg/kg, i.p.), ML171 (60 mg/kg, i.p.), or Veh in *Plp*-cKI-*Trpa1^mut^* or control mice (**B**) fasted, (**D**) exposed to cold, or (**F**) restraint stress. Data are mean ± SEM. (*n* = 8 mice/group) 1-way (**A**–**C**) and 2-way (**E**, **G**, and **I**) ANOVA, Bonferroni correction. ****P < 0.0001 versus Control/Fasted/Veh, Control/Cold/Veh, Control/Restraint/Veh; ^####^*P* < 0.0001 versus *Plp*-cKI-*Trpa1^mut^*/Fasted/Veh, *Plp*-cKI-*Trpa1^mut^*/Cold/Veh, *Plp*-cKI-*Trpa1^mut^*/Restraint/Veh.

## Version 1 10/01/2026

In-Press Preview

## Version 2 11/16/2026

Electronic publication
