# Supplementary material for: Neuronal and nonneuronal TRPA1 mediate distinct pain perception mechanisms in Familial Episodic Pain Syndrome models
Source: J Clin Invest. 2026 Oct 1;136(22):e206667. doi: 10.1172/JCI206667 (PMC13767637; doi:10.1172/JCI206667)
Supplement: Supplementary file 1 [file jci-136-206667-s222.pdf]

Supplemental Material for

Neuronal and non-neuronal TRPA1 mediate distinct pain perception mechanisms in Familial Episodic Pain Syndrome models

Matilde Marini[1]*, Martina Chieca[1]*, Elisabetta Coppi[2]*, Marco Montini[3], Lorenzo Bonacchi[1], Lorenzo Landini[1], Irene Scuffi[1], Kelvin Y Kwan[4], Alice Papini[1], Gaetano De Siena[1], Elisa Bellantoni[1], Lucrezia Timotei[1], Elena Colzi[2], Valentina Albanese[5], Gabriele Ferroni[1], Evelynn Dalila do Nascimento Melo[6], Marie-Christine Birling[7], Romain Lorentz[7], Romina Nassini[1§], Francesco De Logu[1§]

[1]Department of Health Sciences, Clinical Pharmacology and Oncology Section, University of Florence, Florence, 50139, Italy

[2]Department of Neuroscience, Psychology, Drug Research and Child Health (NEUROFARBA), Section of Pharmacology and Toxicology, University of Florence, Florence, 50139, Italy

[3]Department of Experimental and Clinical Biomedical Sciences, University of Florence, Florence, 50139, Italy

[4]Department of Cell Biology & Neuroscience, Keck Center for Collaborative Neuroscience, Stem Cell Research Center, Rutgers University, Piscataway, NJ 08854, USA

[5] Department of Chemical, Pharmaceutical and Agricultural Sciences, University of Ferrara, Via Luigi Borsari 46, 44121 Ferrara, Italy.

[6]Laboratory of Bioactive Products, Federal University of Rio de Janeiro, Multidisciplinary Centre UFRJ-Macaé, Macaé, RJ, Brazil

[7]CNRS, INSERM, CELPHEDIA, PHENOMIN, Institut Clinique de la Souris, UAR2062 – PHEN-ICS

Supplemental methods

Treatment Protocols

If not otherwise indicated, the reagents were obtained from Merck Life Science SRL (Milan, Italy). C57BL/6J mice received intraplantar (i.pl./10 µl site) injection of allyl isothiocyanate (AITC, 0.1, 0.5, 1, 10 nmol) or 4-hydroxynonenal (4-HNE, 0.5, 5, 50 nmol) or their vehicle (1% DMSO in 0.9% NaCl); A967079 (100 mg/kg) or its vehicle (1% DMSO in 0.9% NaCl) were administered intraperitoneal (i.p., 10 ml/kg) 0.5 h before the stimulus. α-Phenyl-N-tert-butylnitrone (PBN, 100 mg/kg, i.p.) its vehicle (2% DMSO in 0.9% NaCl), ML171 (60 mg/kg, i.p.) or its vehicle (4% DMSO, 4% Tween80 in 0.9% NaCl) were administered before and subsequently once daily for two consecutive days in mice subjected to fasting, cold, and three consecutive days in mice subjected to stress.

Behavioural experiments

Acute nociception. Immediately after i.pl. injection, mice were placed inside a plexiglass chamber, and spontaneous nociception was assessed for 10 min by measuring the time (s) that the animal spent licking/lifting the injected paw.

Hindpaw mechanical allodynia. The mechanical paw-withdrawal threshold was measured using von Frey filaments of increasing stiffness (0.02-2 g) applied to the plantar surface of the mouse hind paw, according to the up-and-down paradigm(1). The 50% mechanical paw-withdrawal threshold (g) response was then calculated from the resulting scores.

Fasting schedule. After baseline mechanical threshold assessment, mice were deprived of the standard pellet diet for 48 h. Water was available *ad libitum* throughout the entire experimental period. Behavioral testing was performed every 24 h during the fasting period and after food deprivation. This 48 h fasting protocol was selected to induce a clear differential pain phenotype between wild-type and TRPA1^{mut}-expressing mice, while preserving the absence of fasting-induced mechanical allodynia in control animals.

Single Restraint. Mice were placed in cylindrical tail-access rodent restrainers designed for animals weighing 15-30 g (Stoelting, 51338). Animals were positioned in the restraint devices to prevent movement without impairing normal respiration. Restraint was maintained for 2 h following baseline mechanical threshold assessment. Behavioral testing was conducted 24 h after the restraint time. Control mice were left in their home cages for 2 h without access to food or water to control for the effects of food and water deprivation alone on stress-related response(2). The short-term acute restraint paradigm (2 h), was selected because it represents a subthreshold stress condition that does not induce mechanical allodynia in control mice.

Cold exposure. Mice were housed in plastic cages with *ad libitum* access to food and water and placed in a temperature-controlled environment at 4 ± 2 °C for 14-15 h (overnight). Prior to behavioral testing, all mice were transferred to the experimental room (20-23 °C) and allowed to acclimate for at least 30 min, after which behavioral experiments were performed(3).

Cell lines

HEK293T cell line. Human kidney epithelial (HEK293T) cells (HEK293T; #CRL3216™; ATCC, Manassas, VA, USA RRID:CVCL0063) were cultured in DMEM supplemented with fetal bovine serum (FBS, 10%) and L-glutamine (2 mM) at 37 °C in 5% CO_2 and 95% O_2.

AAVpro 293T cells. AAVpro 293T cells (#632273, Takara), were maintained in DMEM high glucose supplemented with heat inactivated FBS (10%), sodium pyruvate (1 mM), L-glutamine (4 mM) and penicillin/streptomycin (1 mM) in 5% CO_2 and 95% O_2 at 37°C. The day before transfection, cells were plated in DMEM supplemented with FBS (2%).

Primary human Schwann cells (hSCs) Commercial hSCs (hSCs, #P10351, Innoprot, Spain) and were cultured and maintained in Schwann cell medium (#P60123, Innoprot, Spain) at 37 °C in 5% CO_2 and 95% O_2. After 12 passages, cells were discarded and replaced.

Primary mouse Schwann cells (mSCs). mSCs were isolated from sciatic nerves of *Plp-Cre-GEdit*, *Plp-Cre-MiniG*, and *Plp-cKI-Trpa1^{mut}* and Control mice. The epineurium was removed, nerve explants were cut (1 mm segments) and dissociated in HBSS containing hyaluronidase (0.1%) and collagenase (0.05%) for 2 h, at 37 °C in agitation. After centrifugation (150 x g, 10 min, RT), the pellet was resuspended and cultured in DMEM containing streptomycin (100 mg/ml), fetal calf serum (10%), forskolin (2 μM), L-glutamine (2 mM), penicillin (100 U/ml) and neuregulin (10 nM). Cytosine arabinoside (Ara-C, 10 mM) was added after three days, to remove fibroblasts. Cells were cultured at 37 °C in 5% CO2 and 95% O2 for 15 days before experiments by replacing the culture medium every 3 days.

Primary mouse dorsal root ganglion (mDRG) neurons. mDRG neurons were isolated from Adv-Cre-GEdit, Adv-Cre-MiniG, Adv-cKI-Trpa1^{mut} and Control mice. Briefly, DRGs (combined cervical, thoracic, and lumbar) were bilaterally removed using a dissection microscope and then digested enzymatically in HBSS for 35 min at 37 °C using 2 mg/mL of collagenase type 1A and 1 mg/mL of papain. Several passages utilizing a series of syringe needles (23–25G) were used to disrupt ganglia. After centrifuging mouse DRG neurons at 180 x g for 5 min at RT, they were resuspended in DMEM/F12, which contained FBS (10%), penicillin (100 U/ mL), streptomycin (0.1 mg/mL), L-glutamine (2 mM) added with nerve growth factor (100 ng/mL), and cytosine-b-D-arabino-furanoside free base (2.5 mM). Cells were maintained at 37 °C in 5% CO2 and 95% O2 for 3 days before being used.

Reverse transcription-quantitative real time PCR (RT-qPCR). Total RNA was extracted from HEK293T cells and from hSCs using the RNeasy Mini Kit (Qiagen, Hilden, Germany). The quantity and quality of the RNA were assessed by measuring the absorbance at 260 and 280 nm with QIAxpert System. RNA was reverse transcribed using SuperScript™ IV VILO™ Master Mix (Invitrogen by

Thermo fisher Scientific) following the manufacturer's protocol. For relative mRNA quantification, RT-PCR was performed using Rotor Gene® Q software (v.2.3.1.49; Qiagen SpA) and KAPA SYBR® FAST (Sigma Merck). The relative abundance of mRNA transcripts was calculated using the ΔCT method and normalized to glyceraldehyde-3-phosphate dehydrogenase (GAPDH) levels. The sets of primers are reported in Supplementary Table 1.

Plasmids

Plasmids expressing TRPA1 and TRPA1^{mut} for in vitro experiments were purchased from VectorBuilder (pRP CMV 3xFLAG-*mTrpa1*-T2A-Puro VB230123-1262qaf and pRP CMV 3XFLAG-mTRPA1 MUT-T2a-Puro VB230412-1022ruq). To generate mice harbouring mutated copies of the TRPA1 channel, a genome-editing (GEdit) approach and a minigene (MiniG) approach were used. For the GEdit strategy, the plasmid pAAV[Exp]-{Donor_2xsgRNA_TRPA1}-CMV>mCherry (VB230506-1032wnn) was utilized. For the MiniG strategy, the plasmid pAAV[Exp]-{miniGene-DonorMMEJ_gRNA-hTRPA1}-CMV>mCherry (VB230506-1057nhs) was employed. Both approaches were carried out in conjunction with the SaCas9 expression plasmid pAAV[Exp]-{CMV short form}>LL/rev(SaCas9(ns)/T2A/EGFP)/rev(LL) (VB230508-1094rfa). All plasmids were obtained from VectorBuilder.

To generate pAAV[FLEXon]-MPZprom-rev(SaCas9), MPZ promoter was amplify (P1/P2) cut with MluI/SalI (NEB) and cloned into pAAV[FLEXon]-CMV-rev(ORF) replacing CMV promoter. A second step of cloning was used to insert SaCas9 amplified with P3/P4 and cloned into pAAV[FLEXon]-MPZ promoter with NdeI/AscI (NEB). To generate pAAV[Exp]-MPZprom SaCas9, SaCas9 was amplified with P5/P6 and cloned into pAAV-MPZ promoter backbone with SalI/EcoRI (NEB) removing LoxP sites. All plasmid constructs were confirmed by Sanger sequencing. Primer sequences are listed in Supplementary Table 1

AAV Generation

AAV production and cell lysis

Three days before Transfection, AAVpro293T cells were seeded in a CellBIND Polystyrene CellSTACK 2 Chamber (#3310, Corning, Corning, NY, USA) for 48 h to reach 80% confluence. The day before transfection, 250 million cells were seeded in a CellBIND Polystyrene CellSTACK 5 Chamber (#3311; Corning) for 24 h, until 80% of confluence. AAVpro 293T cells were triple transfected with polyethylenimine (#23966, PEI, Polyscience) using a DNA:PEI ratio of 1:3. transfection was performed with 2.5 mg total DNA with 1:1:1 molar ratio of three plasmids (plasmid expressing gene of interest, pAdDeltaF6; (#11287, Addgene) and Rep/Cap plasmid). To infect sensory neurons and Schwann cells with high efficiency, the pAAV2/9n and AAV2/rh10 plasmids were used, respectively (pAAV2/9n, #112865; pAAV Rep2/Cap-rh10, #112866, Addgene). After 72 h, AAV virions were collected. Cells were harvested, transferred to 50 mL conical tubes, and centrifuged at 1200 × g for 10 min at 4 °C. The supernatant was filtered through 0.45 µm PES membrane. To concentrate the virus, 25 mL of 40% PEG solution (pH 7.4; 400 g of PEG 8000 + 24 g of NaCl in ddH$_2$O to a final volume of 1 mL) was added to every 100 mL of filtered supernatant. The solution was slowly stirred at 4 °C for 1 h and then kept for 3 hrs without stirring at 4 °C to allow complete precipitation of AAV particles. After centrifugation at 2900 × g for 15 min at 4 °C, the supernatant was discarded, and the pellet containing virion particles was resuspended in 10 ml of PBS/pluronic F68 (0.001%)/NaCl (200 mM). AAVpro293T cell pellet, derived from the first centrifugation, was directly resuspended in 10 mL of PBS/Pluronic F68 (0.001%)/NaCl (200 mM) solution and lysed by 4 cycles of freezing at -80°C for 30min, followed by a thaw at 37°C for 10 min. After centrifugation at 3.200 × g for 15 min at 4 °C, supernatant containing the AAV particles was collected. Both samples, obtained from the supernatant and cell pellet, were mixed and incubated with Benzonase (50 U/ml) at 37°C for 45 min to remove residual DNA carried over from the AAV packaging process. The sample was then centrifuged at 2.500 × g for 10 min at 4°C, and the clarified supernatant was transferred to a new tube and kept overnight at 4°C before iodixanol purification.

AAV purification by Iodixanol gradient

An iodixanol gradient was prepared. Starting with a 60% iodixanol solution (OptiPrep; STEMCELL Technologies, Vancouver, Canada), 15% iodixanol solution [4.5 mL of iodixanol (60%) + 13.5 mL of NaCl/PBS-MK buffer (1M)], a 25% solution [5 mL of iodixanol (60%) + 7 mL of PBS-MK buffer (1×) + 30 µL of phenol red], a 40% solution [6.7 mL of iodixanol (60%) + 3.3 mL of PBS-MK buffer (1×)], and a 60% solution [10 mL of iodixanol (60%) + 45 µL of phenol red] were made. Each solution was added into a 39-mL Quick-Seal tube (Beckman Coulter, Brea, CA, USA) using an 18 G needle syringe in the following order: 8 mL of 15% iodixanol solution, 6 mL of 25% iodixanol solution, 5 mL of 40% iodixanol solution, and 5 mL of 60% iodixanol solution. Finally, Quick-Seals were filled with AAV solution from the top, sealed, and ultracentrifuged in a Type 70 Ti rotor (Beckman Coulter) at 350.000 x g at 10°C for 1.5 h. Then, Quick-Seal tubes were pierced with a 16 G needle on top and a 14 G needle at the interface between the 60% and 40% iodixanol gradients. Viral particles contained in the 40% iodixanol layer were collected dropwise into 2mL microcentrifuge tubes, combined and concentrated using Amicon Ultra-15 centrifugal filter units with a molecular weight cut-off of 100 kDa (Merck Millipore). Before the concentration step, Amicon membranes were covered with 15 mL of 0.1% Pluronic F68/PBS solution for 10 min, then discarded and replaced with 15 mL of 0.01% Pluronic F68 in PBS. Tubes were centrifuged at 3.000 rpm for 5 min at 4 °C, and the flow-through was discarded. 15 mL of 0.001% Pluronic F68 in PBS + NaCl 200 mM solution was added and centrifuged at 3.000 x rpm for 5 min at 4 °C and the flowthrough was again discarded. During the concentration process, a buffer exchange was performed with the formulation buffer (FB: 0.001% Pluronic F68 in PBS). FB was added to the AAV-iodixanol sample to replace iodixanol and to avoid toxicity in animals after AAV injection. Purified AAV virions were aliquoted and stored at -80°C. The viral titter was quantified using AAVpro® Titration Kit (for Real Time PCR) Ver.2 (Takara). The integrity and uniformity of AAV-packaged vector genomes were analyzed by Oxford Nanopore long-read sequencing. Briefly, purified AAV aliquots were incubated with proteinase K to digest the viral capsid and release protected vector DNA. Recovered AAV genomes were amplified using primers

designed to span the insert-containing regions and the relevant vector junctions. Amplicons covering the expected packaged genome architecture were purified using Gel and PCR Clean-up columns (NucleoSpin®; MACHEREY-NAGEL) according to the manufacturer’s instructions. Purified PCR products were then used to generate multiplexed Oxford Nanopore sequencing libraries using the Ligation Sequencing Amplicons Native Barcoding Kit 24 V14 (SQK-NBD114.24, Oxford Nanopore Technologies), following the manufacturer’s protocol.

Transfection protocol

HEK293T cells were plated in 24-well plates the day before transfection on poly-L-lysine-coated (8.3 μM) 13 mm glass coverslips and maintained at 37°C in 5% CO_2 and 95% O_2. HEK293T cells were transfected with 250 ng of plasmid DNA expressing either mouse wild-type Trpa1 (TRPA1) or the mutated gene ($TRPA1^{mut}$), using Lipofectamine™ 3000 Transfection Reagent (#L3000001, Thermo Fisher) according to the manufacturer's instructions.

Electrophysiology

Whole-cell patch-clamp recordings were performed on 13 mm coated coverslips as previously reported(4, 5). Briefly, coverslips were transferred to a recording chamber (500 μl volume), mounted on the platform of an inverted microscope (Olympus CKX41, Milan, Italy) and superfused at a flow rate of 1.5 $mL \cdot min^{-1}$ with a standard extracellular solution containing (in mM): 10 HEPES, 10 D-glucose, 147 NaCl, 4 KCl, 1 $MgCl_2$ and 2 $CaCl_2$ (pH adjusted to 7.4 with NaOH). Cells were voltage-clamped at -60 mV by 2-4 MW-recording electrodes. HEK-293 transfected cells were recorded with the following intracellular solution (in mM): CsCl 120; Mg_2-ATP3; EGTA10 and HEPES10 (pH 7.4 with CsOH). DRG neurons were recorded with (in mM): K-gluconate 130; NaCl 4.8; KCl 10; MgCl2 2; CaCl2 1; Na2-ATP 2; Na2-GTP 0.3; EGTA 3; and HEPES 10 (pH 7.4 with KOH). All experiments were performed at room temperature (RT: 20-22˚C). Data were acquired with an Axopatch 200B amplifier (Axon Instruments, Union City, CA), low-pass filtered at 10 kHz, stored, and analysed with

pClamp 9.2 software (Axon Instruments). Series resistance (Rs), membrane resistance (Rm), and membrane capacitance (Cm) were routinely measured using fast hyperpolarizing voltage steps of 10 mV. Only cells showing a stable Cm and Rs (<20% variation) throughout the experiment were included in the analysis. A Voltage ramp protocol (-100 to +100 mV; 800 ms) was applied every 15 s to evoke overall voltage-dependent currents in TRPA1- or $TRPA1^{mut}$-transfected HEK293T cells. After a stable baseline was established, AITC (100 μM) or 4-HNE (100 μM) was applied for 2 min, followed by the selective TRPA1 antagonist A967079 (100 μM). TRPA1- or $TRPA1^{mut}$-activated currents were obtained by subtraction of the ramp trace recorded after adding the TRPA1 antagonist A967079 from that recorded in the presence of each agonist. Where indicated, a depolarizing voltage step protocol (-140 to +140 mV, 20 mV increment; 300 ms step duration) was applied before or during agonist exposure. DRG neurons were maintained at -60 mV and currents evoked by AITC (100 μM, 2 min application: I_{AITC}) in the absence or presence of A967079 (100 μM, applied 5 min before, during and after AITC) were recorded. In averaged results, currents were normalized to Cm and expressed as current density (pA/pF).

H_2O_2 *in vitro* imaging

The genetically encoded probe for H_2O_2-HyPer [HyPer7.2(6, 7)] was used on TRPA1 or $TRPA1^{mut}$-HEK293T transfected live cells and on Schwann cells isolated from *Plp-Cre-GEdit, Plp-Cre-MiniG, and Plp-cKI-Trpa1^{mut}* mice. Briefly, cells were plated on poly-L-lysine-coated (8.3 μM) 35-mm glass coverslips and transfected with HyPer7.2 DNA (2 μg) using jetOPTIMUS® DNA transfection reagent (#55-250; Polyplus, Lexington, MA, USA). Cells were washed and transferred to a chamber on the stage of a fluorescent microscope for recording (Axio Observer 7; with a fast filter wheel and Digi-4 lens to record excitations; ZEISS, Stuttgart, Germany) after an incubation period of 24–48 h at 37C° with 5% CO_2 and 95% O_2. Depending on the experiment, cells were exposed hydrogen peroxide (H_2O_2, 3 nM-3 μM), AITC (10 nM-10 μM), 4-HNE (10 nM-10 μM) or vehicle (0.0001% DMSO) and H_2O_2 variations were monitored for approximately 300 s. EC_{80} for H_2O_2 (TRPA1= 809 nM,

$TRPA1^{mut}$= 394 nM), AITC (TRPA1= 1.5 μM, $TRPA1^{mut}$=1.9 μM) and 4-HNE (TRPA1 = 4.4 μM, $TRPA1^{mut}$ = 3.3 μM) was used in the presence of A967079 (30 μM), ML-171 (1 μM) or vehicle (0.0001% DMSO). The $\Delta F/F0_{408/455}$ ratio was calculated for each experiment, and the results were expressed as the area under the curve (AUC).

Immunofluorescence

Anesthetized mice were transcardially perfused with PBS (phosphate-buffered saline), and then tissues (sciatic nerve, spinal cord and dorsal root ganglia, DRG) were collected. Tissues were fixed with 10% formalin overnight at 4°C, and then paraffin-embedded or cryoprotected in 30% sucrose overnight. Frozen samples were cryosectioned at 10 μm or 50 μm while paraffin samples were cut at 5 μm with microtome. Frozen transverse and longitudinal sections (10 μm) of mouse sciatic nerve were incubated with primary antibody 4-HNE (#ab48508, mouse monoclonal (HNEJ-2), Abcam, 1:25, RRID: AB_867461), MBP (#83683, mouse monoclonal (E9P7U), Cell Signaling, 1:400, RRID: AB_2935868) and beta3-Tubulin (#5568, rabbit monoclonal (D71G9), Cell Signaling, 1:400, RRID: AB_10694505) diluted in fresh blocking solution normal goat serum (NGS, 5% in PBS-T) for 1 h at room temperature or overnight at 4°C after incubation with the same blocking solution for 1h at room temperature. Sections were then incubated for 2 h in the dark at room temperature with fluorescent secondary antibody polyclonal Alexa Fluor 647 (#A21236, goat anti-mouse polyclonal, Thermo Fisher Scientific, 1:600, RRID:AB_2535805), Alexa Fluor 488 (#A11001, goat anti-mouse polyclonal, Invitrogen, 1:600, RRID: AB_2534069), Alexa Fluor 647 (#A32733, goat anti-rabbit polyclonal, Invitrogen, 1:600, RRID: AB_2866492) diluted in blocking solution, then washed and coverslipped using a water-based mounting medium with DAPI (#ab104139, Abcam). The 4-HNE staining was evaluated by measuring fluorescence intensity using an image processing program (ImageJ 1.32J, National Institutes of Health). Spinal cord sections (50 μm) were incubated with primary antibody Olig2 (#MABN50, mouse monoclonal (211F1.1), Millipore Merck, 1:200, RRID: AB_10807410), GFP (#A-6455, rabbit polyclonal, Invitrogen, 1:500, RRID: AB_221570) and 4-

HNE (#ab48508, mouse monoclonal (HNEJ-2), Abcam, 1:25, RRID: AB_867461) diluted in blocking solution NGS 5% for 1 h at room temperature or overnight at 4°C after incubation with the same blocking solution for 1h at room temperature. Sections were then incubated for 2 h in the dark at room temperature with fluorescent secondary antibody polyclonal Alexa Fluor 488 (#A32731, goat anti-rabbit, Thermo Fisher Scientific, 1:600, RRID: AB_2633280), Alexa Fluor 594 (#A11005, goat anti-mouse, Invitrogen, 1:600, RRID: AB_2534073) and Alexa Fluor 488 (#A11001, goat anti-mouse polyclonal, Invitrogen, 1:600, RRID: AB_2534069) diluted in blocking solution, then washed and coverslipped using a water-based mounting medium with DAPI.

For formalin-fixed, paraffin-embedded sections, the primary antibody was used after antibody retrieval with citrate buffer (pH 6) and blocking for 1 h with fresh blocking solution containing normal donkey serum (NDS, 5% in PBS-T). Primary antibodies S100 (#ab196175, rabbit monoclonal (EP1576Y), 1:50), NeuN (#MAB377, mouse monoclonal (A60), Merck, 1:400), and GFP-FITC conjugated (#ab6662, goat polyclonal, 1:200) were diluted in NDS 5% and incubated overnight at 4°C. Alexa Fluor 647 (#A21236, goat anti-mouse polyclonal, Thermo Fisher Scientific, 1:600) diluted in NDS 5% was used as a secondary antibody, incubated for 2 h at RT. All slides were mounted with DAPI. All slides were visualized and analyzed using a Zeiss Axio Imager 2 microscope in Z-stack mode (Zeiss). The number of S100- or NeuN-positive cells expressing GFP was manually counted at 600X magnification. We acknowledge that detecting biomarkers localized in two or more intracellular compartments of the same cell may be affected by slice orientation, leading to incomplete colocalization, and that this is a limitation of the method.

Southern blot

All Southern blots were performed using radioactively-labelled probes (α32P) as previously described (8)

Electroporation and Selection of ESCs

Electroporation and screening of ESCs were performed as previously described(9). Briefly, the targeting construct was electroporated using the Gene Pulser Xcell Electroporation System (#165-2661, Bio-Rad, Hercules, CA, USA). In brief, 10 µg of circular targeting vector was combined with 5 × 10^6 ESCs in a 4 mm electroporation cuvette and pulsed at 400 V, 125 µF. Cells were distributed on two 10 cm plates with DR4 feeders (mouse embryonic fibroblasts derived from the Dnmt1tm3Jae Hprtb-m3 Tg(pPWL512hyg)1Ems/J line; (10) in KSR medium. Twenty-four hours after electroporation, the medium was renewed with KSR containing G418 (150 µg/mL). This selection medium was changed every day until clone picking (10 days after electroporation). Picked colonies were trypsinized and triplicated into 96-well plates. One plate was used for the initial screen; the second plate was used to amplify the selected (Long Range-PCR-positive) clones, and the third plate was reserved as a backup. Cells were allowed to grow again in 96-well plates containing feeders for about 4–5 days in KSR medium.

Cell Lysis

Plates were washed with PBS. 75 µL of DirectPCR Lysis Reagent (#302-C Viagen Biotech) and 5 µL proteinase K (10 mg/mL) were added to each well. Plates were wrapped with foil lids and shaken slowly and incubated for 5 h to overnight on a plate shaker at 55°C. The next day, the plates were shaken for a few min to homogenize the lysate. 80 µL of H_2O was added to each well to compensate evaporation. Three wells (one with normal, one with high and one with lower cell content) were kept apart and served as controls for the PCR. In a 96-well plate, 1 µL of each ESC lysate was added to 5 µL of a solution of Tris HCl at 5 mM, and proteinase K was inactivated 5 min at 95°C.

Embryonic Stem Cell Screening and Validation

Long-Range PCR

The gene-specific LR-PCR genotyping primers used to identify clones were designed as follows: primers were selected by examining the genomic sequences flanking the 5′ and 3′ targeting vector homology arms, ensuring they avoided repeated sequences. The 3′ LR-PCR was performed with a gene-specific reverse primer (Rext; 5'-AACAATGTCACCTGCTTGCACTAGC-3') and a forward selection cassette universal primer (Fneo; 5'-AGGGGCTCGCGCCAGCCGAACTGTT-3').

The 5′ LR-PCR is also combined in a mixture with a universal reverse primer (Rneo; 5'-CTGCACGAGACTAGTGAGACGTGC-3') with a gene-specific forward primer (Fext; 5'-GTGTAGGTGTTCAGATCTGTGTTCC-3'). The 5' LoxP-PCR was performed with the same gene-specific forward primer (Fext; 5'-GTGTAGGTGTTCAGATCTGTGTTCC-3') and a LoxP specific reverse primer (Rlox; 5'-CATACATTATACGAAGTTATGGCCG-3').

Screening reactions were performed with a combination of two DNA polymerases: Red Hot Taq polymerase (previously ABGENE #AB-0406/B, special production by Thermo Fisher) and Pwo DNA polymerase (#11644955001, Sigma-Aldrich). Genotyping was carried out on 96 well plates using 20 µL reaction volumes. In each well except the 3 last ones was placed: 2 µL universal or gene-specific forward primer (1.25 µM), 2 µL gene-specific or universal reverse primer (1.25 µM), 6 µL DNA lysate (corresponding to approximately 50 ng), 2 µL 10× buffer, 1.2 µL $MgCl_2$ (25 mM), 0.4 µL dNTP (10 mM), 0.15 µL Red Hot Taq polymerase (5 U/µL), 0.02 µL Pwo DNA polymerase (5 U/µL) and 6.23 µL water. To validate each assay, as no positive control clone was available, the last three wells were kept for control PCR designed as follows: the internal universal primer was replaced with a gene-specific primer (5'-GAGCTGCATGTGTGAATTAAATTCT-3') that was designed at the extremity of the most distal part of the homology arm. The screen was considered valid if at least one of these wells showed a PCR fragment at the expected size. The cycling conditions were as follows: denaturation step of 5 min at 96°C, then 30 cycles of (96°C for 8 s, 60°C for 10 s, 68°C for 6 min)

with an increase of 15 s at each cycle followed by 5 cycles of (96°C for 8 s, 60°C for 10 s, 68°C for 8 min); to finish, the samples were incubated at 68°C for 10 min.

DNA Sequencing Library Preparation, and Analysis

Sciatic nerves were collected from mice and stored in RNA later at -80°C until processing. Total RNA was extracted with RNeasy® Lipid Tissue Mini kit (#74804; Qiagen SpA, Hilden, Germany) according to the manufacturer's protocol. The RNA quantity and quality were determined by measuring the absorbance at 260/280 nm with QIAxpert System. RNA was reverse transcribed using SuperScript™ IV VILO™ Master Mix (Invitrogen) following the manufacturer's protocol. *Wild Type*, CKI and mutated Trpa1 cDNA sequences from exon 18 to exon 24 were amplified with KOD Hot Start DNA Polymerase (#71086, Merck)(primers Fw 5'-TGTCATGGTCCAACATAACCGCA-3'; Rev: 5'-GCATGCTTCTGGACCTCAGC-3'). PCR products were purified using Gel and PCR Clean-up (NucleoSpin®; #740609.50, MACHEREY-NAGEL) according to the manufacturer's instructions. PCR products were used to create a multiplexing library using Ligation sequencing amplicons - Native Barcoding Kit 24 V14 (SQK-NBD114.24, Oxford Nanopore Technologies) according to the manufacturer's protocol. For each sample, 200 fmol of amplicon were used to prepare the library. The first step was end-prep using the NEBNext® Ultra™ II End Repair/dA-Tailing Module (NEB, E7546). This step was followed by barcode ligation and subsequent adapter ligation. The resulting libraries were multiplexed and sequenced on a PromethION 2 Solo device (Oxford Nanopore Technologies) using the MinKNOW software for 12 hours following manufacturer's protocol.

Bioinformatic analysis

Reads were filtered to retain those with a minimum quality score of 10 and a minimum length of 500 base pairs. After quality control (QC) filtering, reads were aligned using the minimap2 tool (version 2.17-r941) with the command option "-ax map-ont", to a custom reference containing the Mus musculus GRCm39 genome and the sequences of the cKI and CTL vectors. Aligned reads were

converted to BAM and sorted with the SAMtools (version 1.10) tool. Coverage of the regions was calculated using mosdepth (version 0.3.12). Following alignment, reads were visualized using IGV software (version 2.19.6). Sequencing yielded a total of 104,360 reads for MiniG, 102,772 for GEdit, and 53,092 for cKI. The percentage of mapped reads was 72% for MiniG (75,797 reads), 71% for GEdit (73,257 reads), and 12% for cKI (6,201 reads). In the region of interest, approximately 6% of reads for both MiniG and GEdit showed the N858S mutation. Additionally, 24 reads were aligned to the cKI vector sequence.

Supplemental figures

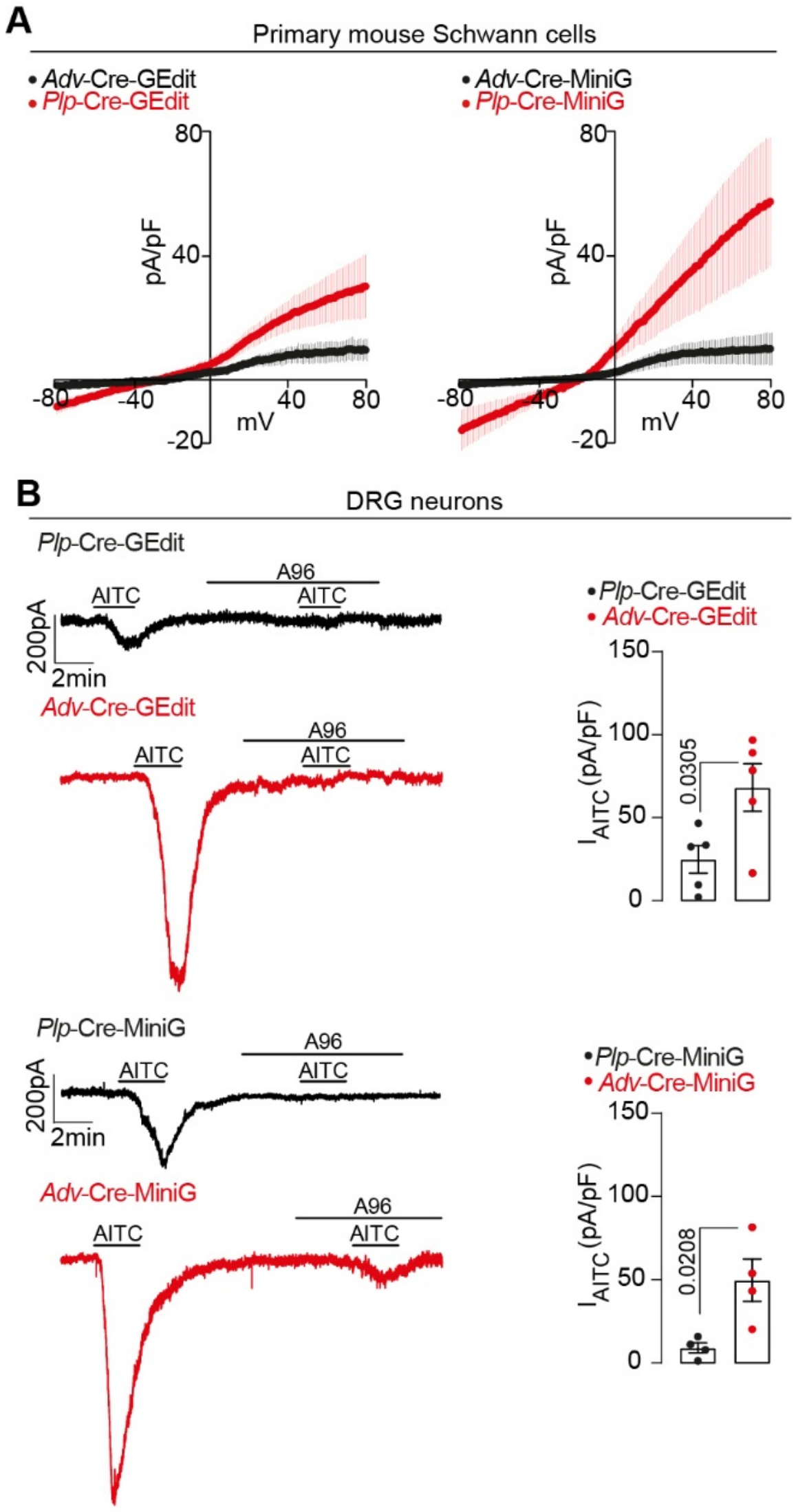

Supplemental Figure 1. Comparison of AITC-activated currents in Schwann cells and DRG neurons from GEdit and MiniG mice. **(A)** AITC (100 µM)-activated currents measured in primary Schwann cells (SCs) from *Plp*-Cre-GEdit (n=7), *Adv*-Cre-GEdit (n=4), *Plp*-Cre-MiniG (n=4), *Adv*-Cre-MiniG (n=7) by subtraction of the control ramp from that recorded in AITC. **(B)** Representative current traces and pooled data of whole-cell patch-clamp inward current in DRG neurons from *Plp*-Cre-GEdit (n=5), *Adv*-Cre-GEdit (n=5), *Plp*-Cre-MiniG (n=4), *Adv*-Cre-MiniG (n=4) stimulated with AITC (100 µM) and in the presence of A967079 (A96, 100 µM). Data are mean ± s.e.m. 2-tailed Student't test (**B**).

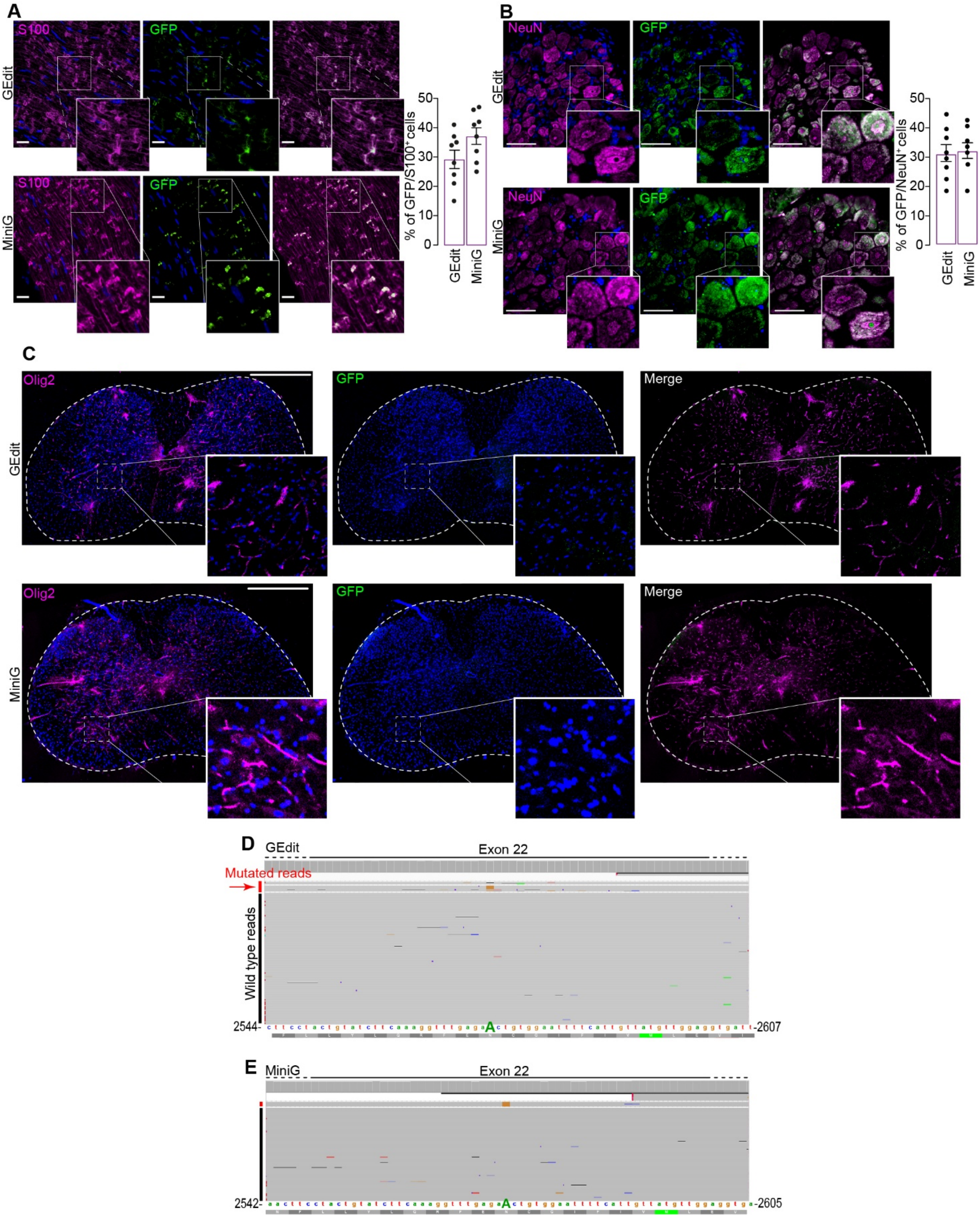

Supplemental Figure 2. *Trpa1^{mut}* Nanopore sequence alignments in GEdit and MiniG mice. Representative images and cumulative data (% of eGFP positive cells) in GEdit and MiniG approaches efficiency expressed as eGFP positive cells in **(A)** mouse sciatic nerve and **(B)** primary sensory neurons (scale bar: 10 µm). eGFP expression is associated with saCas9 expression. Data are

mean ± s.e.m. (**C**) Representative images of spinal cord sections of GEdit and MiniG mice showing Olig2 and GFP staining (scale bar: 500 µm). Integrated Genome Viewer (IGV version 2.19.6) visualization of long-read alignments from **(D)** GEdit and **(E)** MiniG mice. The reference sequence (color letters) represents the vector sequence, with the *Trpa1* wild-type base (**A**) shown in uppercase. Mutated base (**G**) for *Trpa1*^{mut} is highlighted in orange.

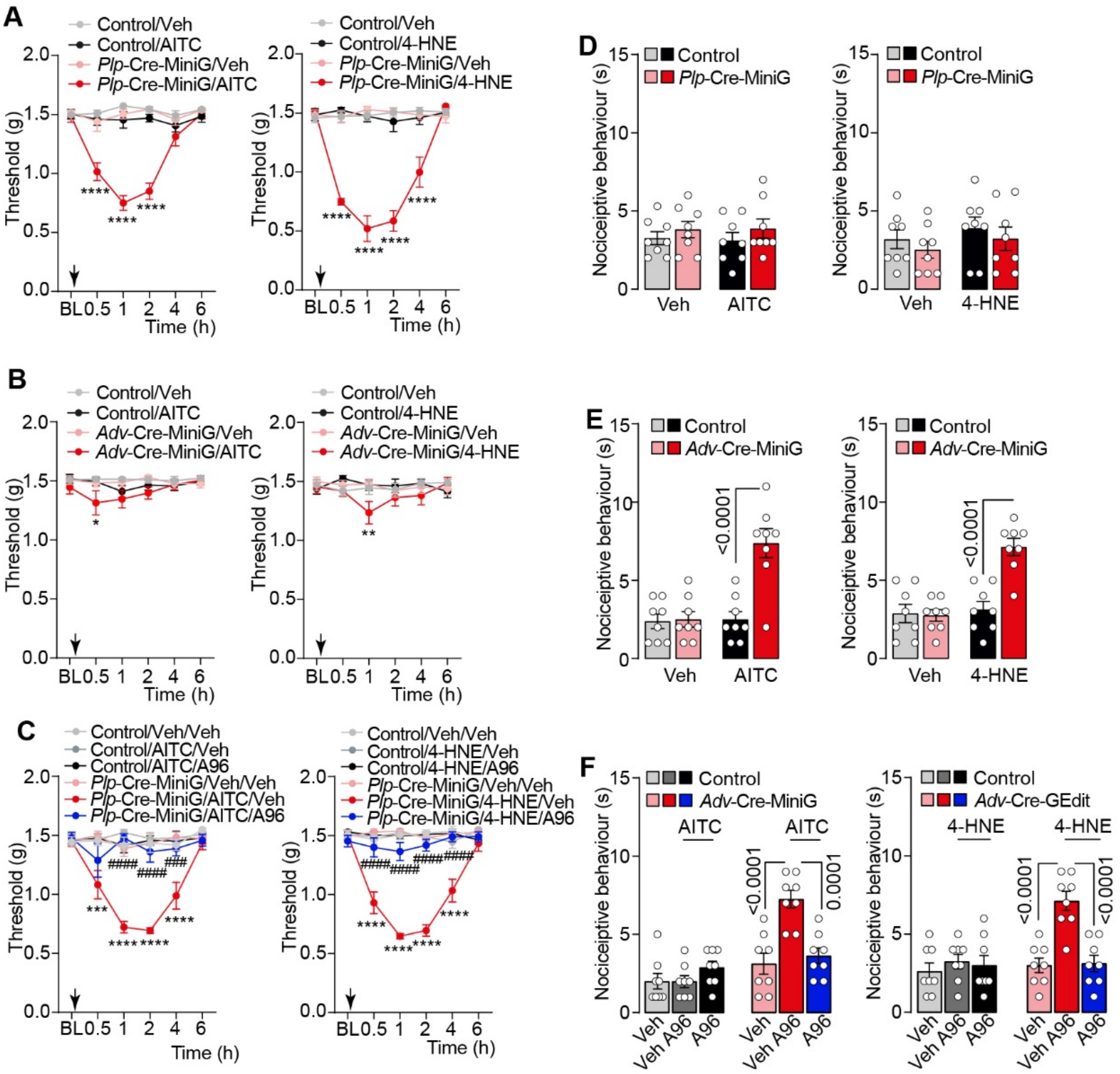

Supplemental Figure 3. Allodynia and non-evoked nociception in mouse with mutated TRPA1 in Schwann cells and primary sensory neurons. (A,B) Mechanical allodynia after intraplantar (i.pl./10 μl) injection of AITC (0.1 nmol), 4-HNE, (0.5 nmol) or vehicle (Veh) in *Plp*-Cre-MiniG, *Adv*-Cre-MiniG or Control mice. **(C)** Mechanical allodynia after i.pl. AITC (0.1 nmol), 4-HNE, (0.5 nmol) or Veh in *Plp*-Cre-MiniG mice pretreated with A967079 (A96, 100 mg/kg, intraperitoneal, i.p.) or Veh. **(D,E)** Acute nociception after i.pl. AITC AITC (0.1 nmol), 4-HNE, (0.5 nmol) or Veh in *Plp*-Cre-MiniG, *Adv*-Cre-MiniG or Control mice. **(F)** Acute nociception after i.pl. AITC (0.1 nmol), 4-HNE, (0.5 nmol) or Veh in *Adv*-Cre-MiniG, *Adv*-Cre-GEdit mice pretreated with A96 (100 mg/kg, i.p.) or Veh. Data are mean ± s.e.m. (n=8 mice/group). Arrow indicates time of compounds administration. 1-way (**D-F**) and 2-way (**A-C**) ANOVA, Bonferroni correction. *p<0.05, **p<0.01, ***p<0.001, ****p<0.0001 vs Control/Veh, Control/Veh/Veh; #p<0.05, ##p<0.01, ###p<0.001, ####p<0.0001 vs *Plp-Cre*-MiniG/Veh/Veh.

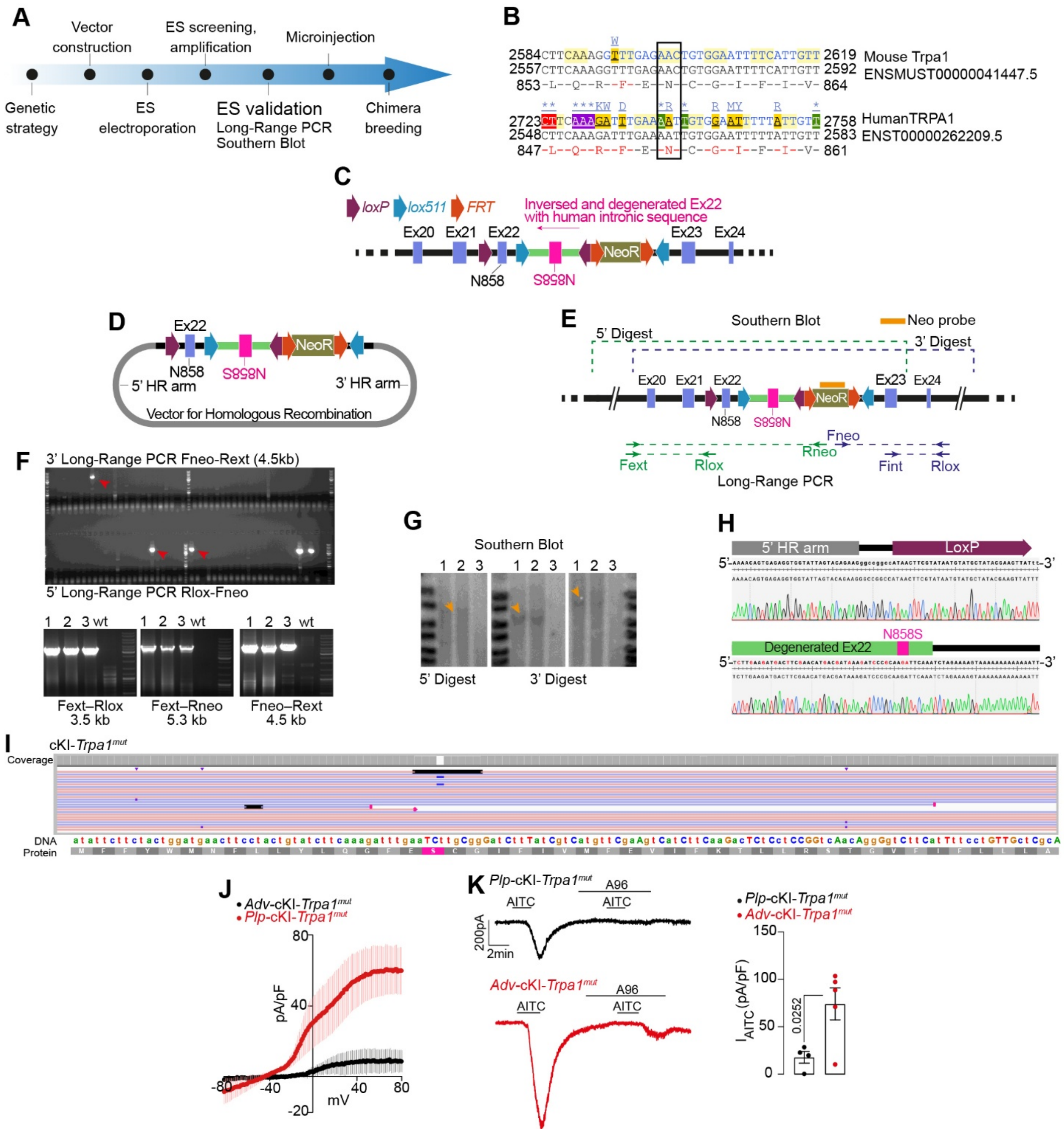

Supplemental Figure 4. Generation of a conditional knock-in (cKI) mouse model carrying a heterozygous FEPS mutation. (A) Experimental timeline and workflow for development of the cKI mouse model. **(B)** Partial sequences of mouse *Trpa1* and human *TRPA1*, highlighting DNA triplet encoding Asparagine. **(C)** Schematic overview of the genetic strategy used to generate the FEPS cKI mouse model. **(D)** Targeting vector containing the cKI cassette flanked by homology arms for electroporation into mouse embryonic stem (ES) cells. **(E)** Schematic representation of 5′ and 3′ long-range PCR and Southern blot analysis using a Neo probe. **(F)** Screening of recombinant ES clones by 3′ long-range PCR (red arrow) and subsequent validation by 5′ long-range PCR of the three candidate clones identified. **(G)** Southern blot analysis following 5′ and 3′ enzymatic digestions using HindIII and SacI or PacI, respectively; orange arrows indicate the expected bands. **(H)** Validation of the 5′ *LoxP* site (top) and the inverted, degenerated exon 22 containing human intronic sequence by Sanger sequencing (bottom). **(I)** Integrated Genome Viewer (IGV version 2.19.6) visualization of long-read alignment from cKI mice. The reference sequence (color letters) represents the vector sequence, with

degenerated bases shown in uppercase. **(J)** AITC (100 μM)-activated currents measured in primary Schwann cells (SCs) from *Plp*-cKI- *Trpa1*^{mut} (n=4) and *Adv*-cKI- *Trpa1*^{mut} (n=5), by subtraction of the control ramp from that recorded in AITC. **(K)** Representative current traces and pooled data of whole-cell patch-clamp inward current in DRG neurons from *Plp*-cKI- *Trpa1*^{mut} (n=4) and *Adv*-cKI- *Trpa1*^{mut} (n=5) stimulated with AITC (100 μM) and in the presence of A967079 (A96, 100 μM). Data are mean ± s.e.m. 2-tailed Student't test (**K**).

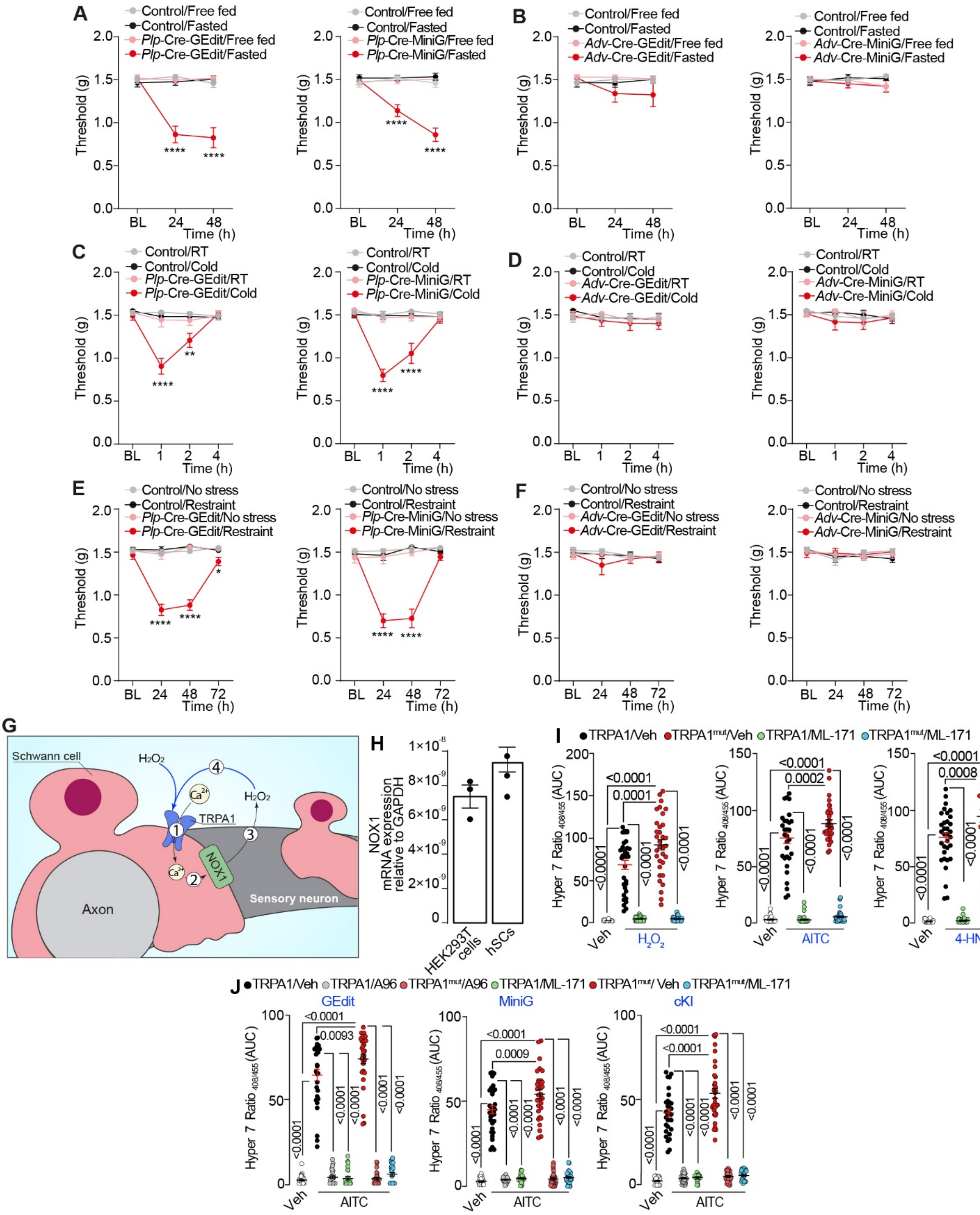

Supplemental Figure 5. Fasting, cold exposure, and restraint stress induce Schwann cell-dependent mechanical allodynia through mutant TRPA1 and a NOX1-mediated feed-forward oxidative mechanism. (A,B) Time dependent mechanical allodynia in Free fed or Fasted *Plp*-Cre-GEdit, *Plp*-Cre-MiniG, *Adv*-Cre-GEdit, *Adv*-Cre-MiniG or Control mice. **(C,D)** Time dependent mechanical allodynia exposed to cold or room temperature (RT) in *Plp*-Cre-GEdit, *Plp*-Cre-MiniG, *Adv*-Cre-GEdit, *Adv*-Cre-MiniG or Control mice. **(E,F)** Time dependent mechanical allodynia in the

presence or absence of restraint stress in *Plp*-Cre-GEdit, *Plp*-Cre-MiniG, *Adv*-Cre-GEdit, *Adv*-Cre-MiniG or Control mice. (n=8 mice/group). **(G)** Proposed feed-forward mechanism of H_2O_2 production and amplification in Schwann cells: (1) H_2O_2 induces TRPA1-dependent Ca^{2+} influx in Schwann cells; (2) the increase in intracellular Ca^{2+} promotes NADPH oxidase 1 (NOX1) activation and (3) subsequent H_2O_2 production; (4) NOX1-derived H_2O_2 further sustains this feed-forward loop, promoting the maintenance and amplification of H_2O_2 release from Schwann cells. **(H)** RT-qPCR for NOX1 mRNA in HEK293T and commercial human primary Schwann cells (hSCs). **(I)** pooled data of HyPer7.2 in HEK293T cells transfected with the mouse TRPA1 channel (TRPA1) or its mutated counterpart ($TRPA1^{mut}$), stimulated with H_2O_2, AITC and 4-HNE in the presence of ML-171 (1 μM) or vehicle (Veh, 0.0001% DMSO) (cells number: H_2O_2 cumulative data: Veh n=37, TRPA1/Veh n=37, TRPA1/ML-171 n=37, $TRPA1^{mut}$/Veh n=36, $TRPA1^{mut}$/ ML-171 n=37; AITC cumulative data: Veh n=34, TRPA1/Veh n=31, TRPA1/ML-171 n=40, $TRPA1^{mut}$/Veh n=30, $TRPA1^{mut}$/ML-171 n=37; 4-HNE cumulative data: Veh n=29, TRPA1/Veh n=33, TRPA1/ML-171 n=31, $TRPA1^{mut}$/Veh n=35, $TRPA1^{mut}$/ ML-171 n=35, 3 independent experiments). **(J)** pooled data of HyPer7.2 in primary mouse Schwann cells (mSCs) from *Plp-Cre-GEdit*, *Plp-Cre-MiniG*, and *Plp-cKI-Trpa1*^{mut} mice, stimulated with AITC in the presence of A967079 (A96, 30 μM), ML-171 (1 μM) or Veh (0.0001% DMSO) (cells number: *Plp-Cre-MiniG* cumulative data: Veh n=34, TRPA1/Veh n=33, TRPA1/A96 n=35, TRPA1/ML-171 n=31, $TRPA1^{mut}$/Veh n=30, $TRPA1^{mut}$/A96 n=34, $TRPA1^{mut}$/ML-171 n=33; *Plp-Cre-GEdit* cumulative data: Veh n=29, TRPA1/Veh n=27, TRPA1/A96 n=25, TRPA1/ML-171 n=27, $TRPA1^{mut}$/Veh n=29, $TRPA1^{mut}$/A96 n=25, $TRPA1^{mut}$/ML-171 n=27; *Plp-cKI-Trpa1*^{mut} mice cumulative data: Veh n=31, TRPA1/Veh n=30, TRPA1/A96 n=33, TRPA1/ML-171 n=35, $TRPA1^{mut}$/Veh n=34, $TRPA1^{mut}$/A96 n=31, $TRPA1^{mut}$/ML-171 n=35). Data are mean ± s.e.m. 1-way (**I,J**), 2-way (**A-F**) ANOVA, Bonferroni correction. **p<0.01, ****p<0.0001 vs Control/Fasted, Control/Cold, Control/Restraint.

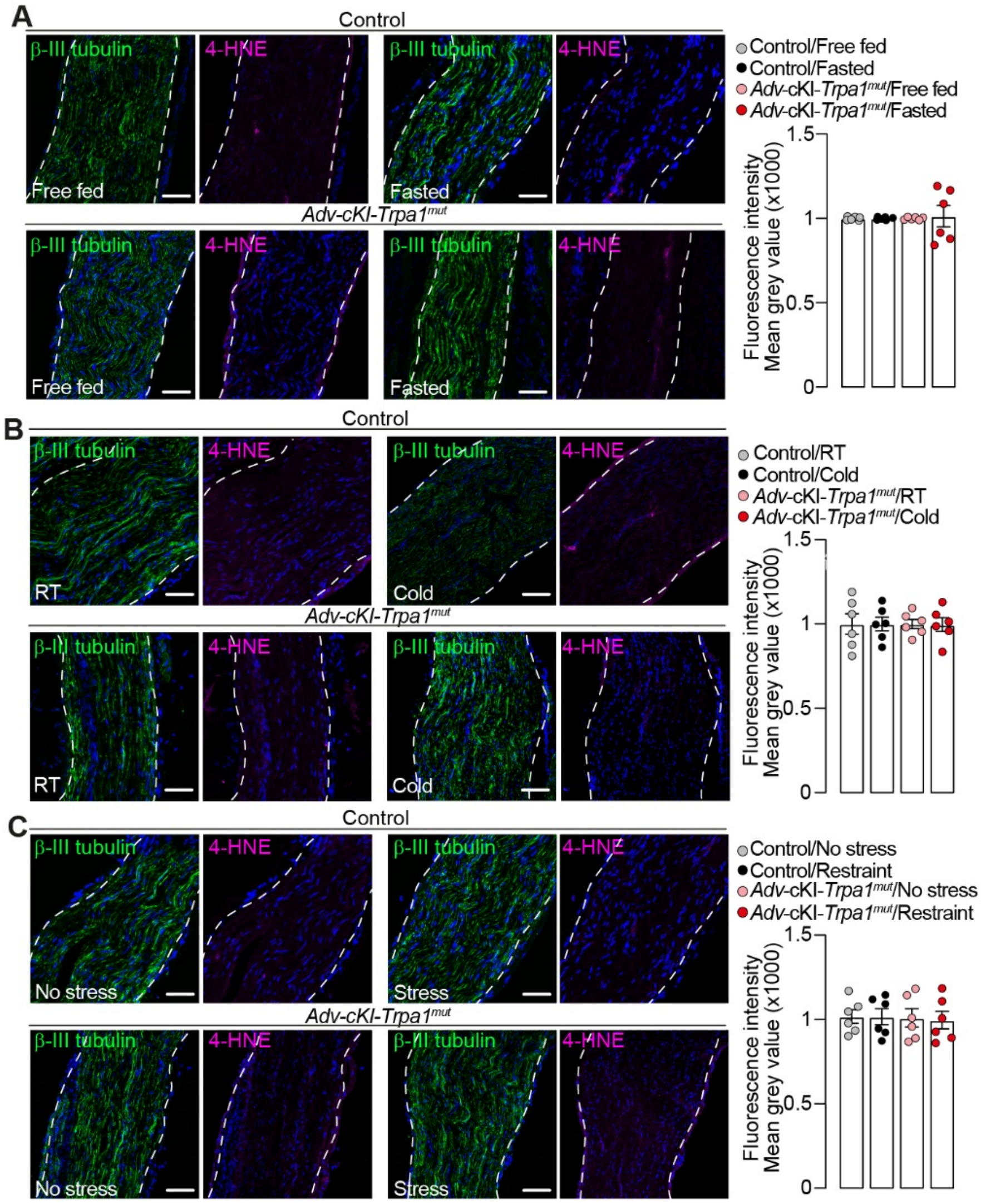

Supplemental Figure 6. Mutant TRPA1 channel generates oxidative stress to maintain mechanical allodynia. Representative images and cumulative data of 4-HNE content within the sciatic nerve of *Adv*-cKI-*Trpa1^{mut}* or Control mice **(A)** free fed or fasted **(B)** exposed to cold or RT or **(C)** in the presence or absence of restraint stress. (n=6, scale bar: 50 µm). β-III tubulin was used as axon-marker. Data are mean ± s.e.m. 1-way ANOVA (**A-C**), Bonferroni correction. Dashed lines indicate the epineurium.

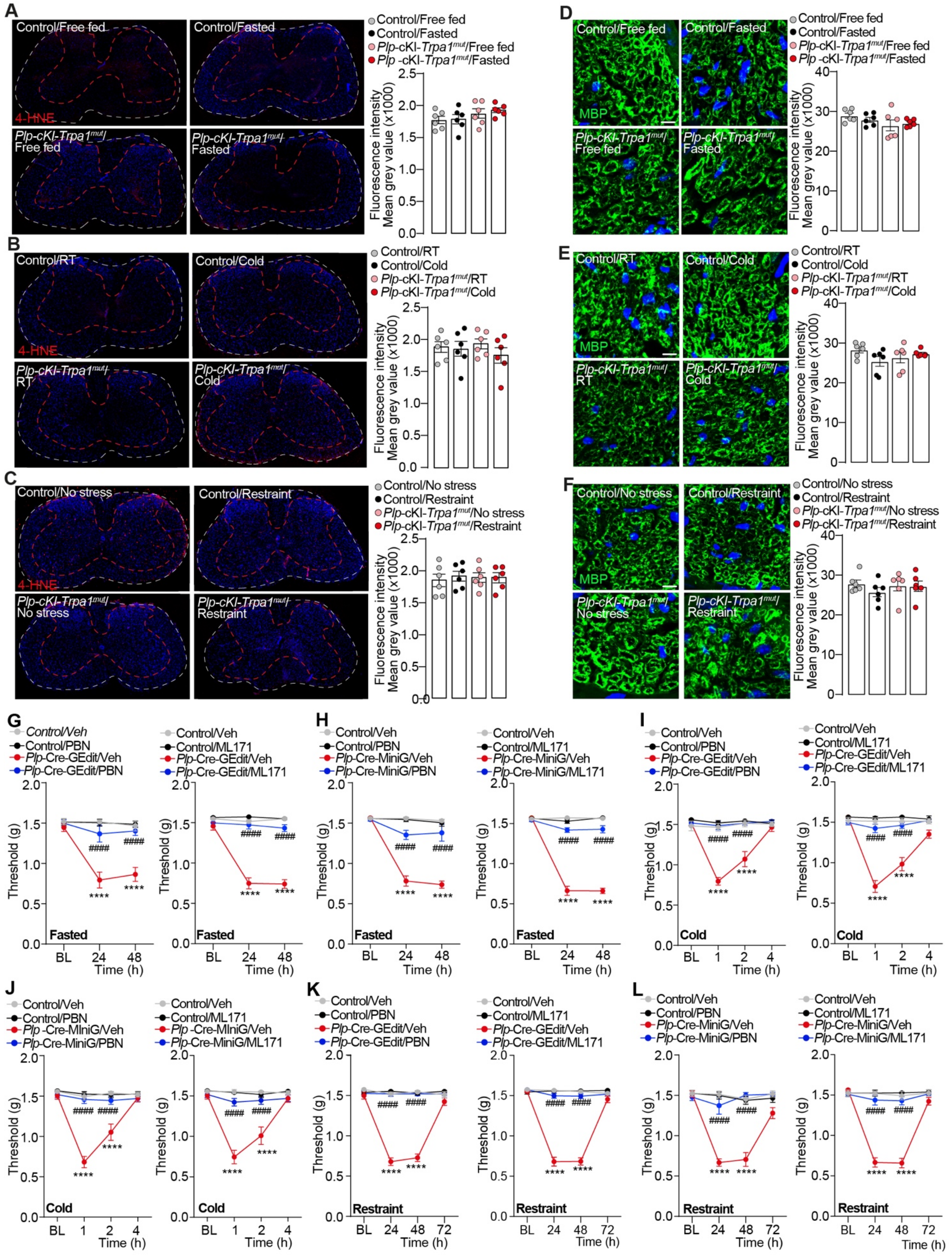
A
Control/Free fed
Control/Fasted
Plp-cKI-Trpa1mut/
Free fed
Plp-cKI-Trpa1mut/
Fasted
4-HNE
Control/Free fed
Control/Fasted
Plp-cKI-Trpa1mut/Free fed
Plp -cKI-Trpa1mut/Fasted
Fluorescence intensity
Mean grey value (x1000)
B
Control/RT
Control/Cold
Plp-cKI-Trpa1mut/
RT
Plp-cKI-Trpa1mut/
Cold
4-HNE
Control/RT
Control/Cold
Plp-cKI-Trpa1mut/RT
Plp-cKI-Trpa1mut/Cold
C
Control/No stress
Control/Restraint
Plp-cKI-Trpa1mut/
No stress
Plp-cKI-Trpa1mut/
Restraint
4-HNE
Control/No stress
Control/Restraint
Plp-cKI-Trpa1mut/No stress
Plp-cKI-Trpa1mut/Restraint
D
MBP
E
F
G
Control/Veh
Control/PBN
Plp-Cre-GEdit/Veh
Plp-Cre-GEdit/PBN
Control/ML171
Plp-Cre-GEdit/ML171
Threshold (g)
Fasted
Time (h)
H
Plp-Cre-MiniG/Veh
Plp-Cre-MiniG/PBN
Plp-Cre-MiniG/ML171
I
Cold
J
K
Restraint
L
****
####

Supplemental Figure 7. Mutant TRPA1 channel generates oxidative stress to maintain mechanical allodynia. Representative images and pooled data of 4-HNE content within spinal cord sections of *Plp*-cKI-*Trpa1^{mut}* or Control mice **(A)** free fed or fasted **(B)** exposed to cold or RT or **(C)** in the presence or absence of restraint stress. (n=6, scale bar: 10 μm). Dashed lines indicate the edges of white matter (white) and gray matter (red). Representative images and cumulative data of myelin basic protein (MBP) within the sciatic nerve sections of *Plp*-cKI-*Trpa1^{mut}* or Control mice **(D)** free fed or fasted **(E)** exposed to cold or RT or **(F)** in the presence or absence of restraint stress. Time dependent mechanical allodynia following repeated administration of phenyl-N-tert-butylnitrone (PBN, 100 mg/kg,intraperitoneal, i.p.), ML171 (60 mg/kg, i.p.) or vehicle (Veh) in *Plp*-Cre-GEdit, *Plp*-Cre-MiniG or Control mice **(G,H)** free fed or fasted, **(I,J)** exposed to cold or RT or **(K,L)** in the presence or absence of restraint stress. Data are mean ± s.e.m. (n=8 mice/group). 1-way (**A-F**), 2-way (**G-L**) ANOVA, Bonferroni correction. ****p<0.0001 vs Control/Fasted, Control/Cold, Control/Restraint, ####p<0.0001 vs *Plp*-Cre-GEdit/Fasted/Veh, *Plp*-Cre-MiniG/Fasted/Veh, *Plp*-Cre-GEdit/Cold/Veh, *Plp*-Cre-MiniG/Cold/Veh, *Plp*-Cre-GEdit/Restraint/Veh or *Plp*-Cre-MiniG/ Restraint /Veh.

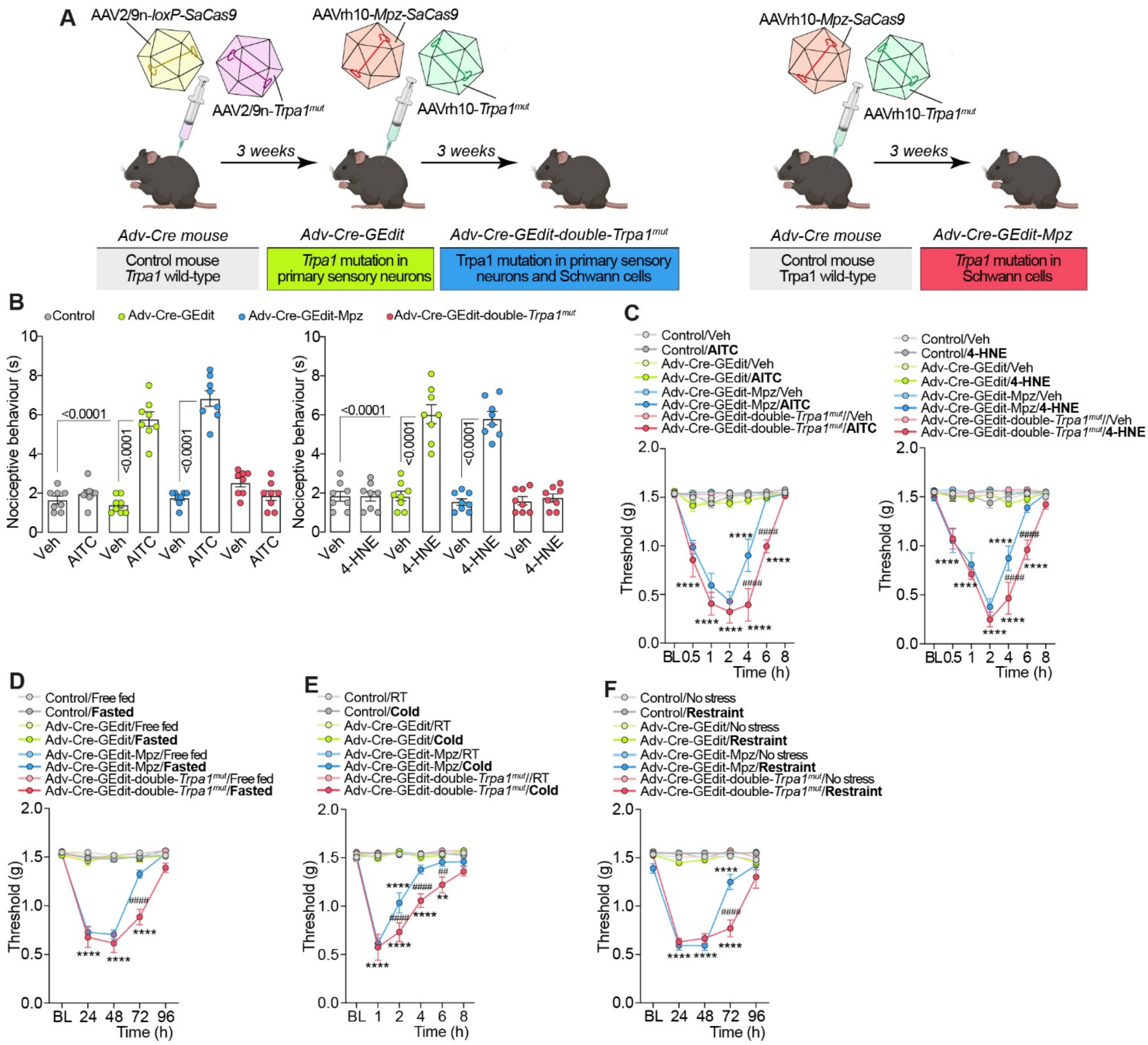

Supplemental Figure 8. Schwann cell and neuronal TRPA1^{mut} contribute to mechanical allodynia. (A) schematic illustration of mice with TRPA1 mutation in Schwann cells and primary sensory neurons. Adv-Cre mice received an AAV2/9n vector carrying a Cre-dependent, loxP-flanked SaCas9 cassette, thereby restricting SaCas9 expression and genome editing to Cre-expressing primary sensory neurons *(Adv*-Cre-GEdit). After 3 weeks same mice received an AAVrh10 vector carrying a non-Cre-dependent SaCas9 cassette under the control of the Schwann cell-selective Mpz promoter (*Adv*-Cre-GEdit-double-*Trpa1^{mut}*). Control mice were infected with AAVrh10-*Mpz-Sa Cas9* (*Adv*-Cre-GEdit-*Mpz)*. **(B)** Acute nociceptive response after intraplantar (i.pl./10 µl) injection of AITC (0.1 nmol), 4-HNE (0.5 nmol) or vehicle (Veh) in *Adv*-Cre-GEdit-double-*Trpa1^{mut}*, Adv-Cre-GEdit, *Adv*-Cre-GEdit-*Mpz* or Control mice. **(C)** Time dependent mechanical allodynia after i.pl. AITC (0.1 nmol), 4-HNE (0.5 nmol) or Veh in *Adv*-Cre-GEdit-double-*Trpa1^{mut}*, Adv-Cre-GEdit, *Adv*-Cre-GEdit-*Mpz* or Control mice. Time dependent mechanical allodynia *Adv*-Cre-GEdit-double-*Trpa1^{mut}*, Adv-Cre-GEdit, *Adv*-Cre-GEdit-*Mpz* or Control mice **(D)** free fed or fasted, **(E)** exposed to cold or room temperature (RT) or **(F)** in the presence or absence of restraint stress**.** Data are mean ± s.e.m. (n=8 mice/group). 1-way (**B**), 2-way (**E-F**) ANOVA, Bonferroni correction. ****p<0.0001 vs

Control/AITC, Control/4-HNE, Control/Fasted, Control/Cold, Control/Restraint, $^{##}p<0.01$, $^{####}p<0.0001$ vs *Adv*-Cre-GEdit-*Mpz*/Fasted, *Adv*-Cre-GEdit-*Mpz*/Cold, *Adv*-Cre-GEdit-*Mpz*/Restraint.

Supplemental Table 1: Primers for plasmids construction and RT-qPCR

PRIMER	SEQUENCE 5’-3’
P1	AAACGCGTGAGCTCTTCACCCTCT
P2	CGTGTCGACTAGCCCATAGCTGGGGTAG
P3	AGCCATATGATGGCCCCAAAGAAGAAGCG
P4	AAAGGCGCGCCTTAAGCGTAATCTGGAACAT
P5	ACTGTCGACATGGCCCCAAAGAAGAA
P6	CGCGAATTCCTAAGCGTAATCTGGAACATCG
NOX1	F: 5’-TTGGGTCAACATTGGCCTGT-3’<br>R: 5’-AAGGACAGCAGATTGCGACA-3’

References

1. Chaplan SR, Bach FW, Pogrel JW, Chung JM, and Yaksh TL. Quantitative assessment of tactile allodynia in the rat paw. *J Neurosci Methods.* 1994;53(1):55-63.
2. Avona A, Mason BN, Lackovic J, Wajahat N, Motina M, Quigley L, et al. Repetitive stress in mice causes migraine-like behaviors and calcitonin gene-related peptide-dependent hyperalgesic priming to a migraine trigger. *Pain.* 2020;161(11):2539-50.
3. Matsubara Y, Okuda H, Harada KH, Youssefian S, and Koizumi A. Mechanical allodynia triggered by cold exposure in mice with the Scn11a p.R222S mutation: a novel model of drug therapy for neuropathic pain related to Na(V)1.9. *Naunyn Schmiedebergs Arch Pharmacol.* 2021;394(2):299-306.
4. Benemei S, De Logu F, Li Puma S, Marone IM, Coppi E, Ugolini F, et al. The anti-migraine component of butterbur extracts, isopetasin, desensitizes peptidergic nociceptors by acting on TRPA1 cation channel. *Br J Pharmacol.* 2017;174(17):2897-911.
5. Marini M, Landini L, Coppi E, Tesi M, Bellantoni E, Chieca M, et al. Anti-nociceptive action of leonurine through TRPA1 and TRPV4 channels modulation. *Br J Pharmacol.* 2025;182(22):5469-88.
6. Landini L, Marini M, Souza Monteiro de Araujo D, Romitelli A, Montini M, Albanese V, et al. Schwann cell insulin-like growth factor receptor type-1 mediates metastatic bone cancer pain in mice. *Brain Behav Immun.* 2023;110:348-64.
7. Pak VV, Ezerina D, Lyublinskaya OG, Pedre B, Tyurin-Kuzmin PA, Mishina NM, et al. Ultrasensitive Genetically Encoded Indicator for Hydrogen Peroxide Identifies Roles for the Oxidant in Cell Migration and Mitochondrial Function. *Cell Metab.* 2020;31(3):642-53 e6.
8. Codner GF, Erbs V, Loeffler J, Chessum L, Caulder A, Jullien N, et al. Universal Southern blot protocol with cold or radioactive probes for the validation of alleles obtained by homologous recombination. *Methods.* 2021;191:59-67.

9. Erbs V, Lorentz R, Eisenman B, Schaeffer L, Luppi L, Lindner L, et al. Increased On-Target Rate and Risk of Concatemerization after CRISPR-Enhanced Targeting in ES Cells. *Genes (Basel)*. 2023;14(2).

10. Tucker KL, Wang Y, Dausman J, and Jaenisch R. A transgenic mouse strain expressing four drug-selectable marker genes. *Nucleic Acids Res.* 1997;25(18):3745-6.
